# BrnQ-Type Branched-Chain Amino Acid Transporters Influence Bacillus anthracis Growth and Virulence

**DOI:** 10.1128/mbio.03640-21

**Published:** 2022-01-25

**Authors:** Soumita Dutta, Ileana D. Corsi, Naomi Bier, Theresa M. Koehler

**Affiliations:** a Department of Microbiology and Molecular Genetics, McGovern Medical School, The University of Texas Health Science Center at Houston, Houston, Texas, USA; b MD Anderson UTHealth Graduate School of Biomedical Sciences, The University of Texas, Houston, Texas, USA; University of Oklahoma Health Sciences Center

**Keywords:** *Bacillus*, amino acid transport, anthracis, anthrax, branched-chain amino acid, nutritional immunity, virulence regulation

## Abstract

Bacillus anthracis, the anthrax agent, exhibits robust proliferation in diverse niches of mammalian hosts. The metabolic attributes of B. anthracis that permit rapid growth in multiple mammalian tissues have not been established. We posit that branched-chain amino acid (BCAA) (isoleucine, leucine, and valine) metabolism is key to B. anthracis pathogenesis. Increasing evidence indicates the relationships between B. anthracis virulence and the expression of BCAA-related genes. The expression of some BCAA-related genes is altered during culture in bovine blood *in vitro*, and the bacterium exhibits valine auxotrophy in a blood serum mimic medium. Transcriptome analyses have revealed that the virulence regulator AtxA, which positively affects the expression of the anthrax toxin and capsule genes, negatively regulates genes predicted to be associated with BCAA biosynthesis and transport. Here, we show that B. anthracis growth in defined medium is severely restricted in the absence of exogenous BCAAs, indicating that BCAA transport is required for optimal growth *in vitro*. We demonstrate functional redundancy among multiple BrnQ-type BCAA transporters. Three transporters are associated with isoleucine and valine transport, and the deletion of one, BrnQ3, attenuates virulence in a murine model for anthrax. Interestingly, an *ilvD*-null mutant lacking dihydroxy acid dehydratase, an enzyme essential for BCAA synthesis, exhibits unperturbed growth when cultured in medium containing BCAAs but is highly attenuated in the murine model. Finally, our data show that BCAAs enhance AtxA activity in a dose-dependent manner, suggesting a model in which BCAAs serve as a signal for virulence gene expression.

## INTRODUCTION

Bacillus anthracis, the bacterium that causes anthrax, is well known for its robust proliferation in diverse niches of mammalian hosts. Infection can result in up to 10^8^ CFU per g of tissue, including various organs, blood, and cerebral spinal fluid, at the time of host death (1–5). While the roles of several secreted virulence factors, including the anthrax toxin proteins, poly-d-glutamic acid capsule, siderophores, and proteases, have been discerned for anthrax (2, 6–9), the metabolic attributes of B. anthracis that permit rapid proliferation to high numbers in multiple mammalian tissues have not been established. B. anthracis is a facultative anaerobe and grows in most rich undefined media with a doubling time of approximately 30 min. Defined minimal media that support B. anthracis growth *in vitro* contain glucose, salts, and nine or more amino acids, but reports differ regarding essential nutrients (10, 11). The B. anthracis genome reveals a repertoire of metabolic and transport genes that are homologous to genes of the well-studied and nonpathogenic *Bacillus* species B. subtilis (12, 13). Yet remarkably, genomic and transcriptomic analyses indicate that B. anthracis has a large capacity for amino acid and peptide utilization, compared to B. subtilis (4, 12, 14, 15). Here, we explore branched-chain amino acid (BCAA) metabolism by B. anthracis as a potential key aspect of anthrax pathogenesis.

The BCAAs, isoleucine, leucine, and valine, are required for protein synthesis and serve as precursors of branched-chain fatty acids, the major fatty acids of the Gram-positive bacterial cell membrane (16). BCAAs can also act as signals related to nutritional status. For many low G+C-content Gram-positive bacteria, BCAAs are effectors for the global transcriptional regulator CodY, which modulates gene expression to support environmental adaptation (17, 18). In some pathogens, BCAA biosynthesis, BCAA transport, or both processes can affect virulence (16, 19–22). BCAAs, like all amino acids, can be synthesized from intermediates of central metabolic pathways if the bacterium possesses the appropriate biosynthetic enzymes or can be transported into the bacterial cell from the environment if the cell envelope includes the proper transport machinery.

The BCAA biosynthesis pathway is highly conserved among bacteria (23). The pathway is composed of eight enzymes, some of which are involved in the biosynthesis of multiple BCAAs and some of which are BCAA specific. Interestingly, despite possessing all enzymes required for BCAA biosynthesis, culture of some bacteria, including Listeria monocytogenes, Streptococcus pneumoniae, and Streptococcus suis, requires the presence of BCAAs (24–26). Others, such as Staphylococcus aureus, show significantly delayed growth in media deficient for one or more BCAAs (27).

The acquisition of BCAAs from the environment can be accomplished by some bacteria that use specialized transporters to bring BCAAs into the cell cytosol. Putative BCAA transporter genes are commonly found in bacterial genomes; however, transporters have been characterized in only a few species (16, 28–30). BCAA transport has been described most extensively in B. subtilis, Lactococcus lactis, and the pathogen S. aureus. In B. subtilis, BrnQ, BcaP, and BraB are major transporters for isoleucine and valine and are likely also able to transport leucine (29). In S. aureus, BrnQ1, BrnQ2, and BcaP have been characterized as BCAA transporters (16, 21). Two BCAA transporters with redundant function have been reported for L. lactis (31, 32), while Corynebacterium glutamicum and Lactobacillus delbrueckii appear to each carry only one BCAA transporter (28, 30).

BCAA synthesis and transport have not been well studied in B. anthracis; however, some reports suggest that BCAA metabolism is related to B. anthracis pathogenesis. B. anthracis exhibits valine auxotrophy when cultured in a blood serum mimic (BSM) medium (33). When B. anthracis is grown in bovine blood *in vitro*, genes for putative BrnQ-related transporters are highly induced, whereas BCAA biosynthesis-related genes and other BCAA-associated ABC transporter genes are repressed (34). Perhaps most intriguingly, the transcript levels of *brnQ*-related transport genes and predicted BCAA biosynthesis genes respond to host-related cues during culture and are regulated by AtxA, a critical regulator of virulence in B. anthracis. AtxA positively affects the expression of the anthrax toxin and capsule genes (35–37) and is essential for virulence in some animal models for anthrax (36, 38). Recently, McCall and coworkers demonstrated the specific binding of AtxA to the *pagA* promoter region (39). While the precise mechanism for AtxA function is not clear, we have demonstrated that AtxA activity and multimerization are enhanced by host-related signals, including CO_2_/bicarbonate and glucose (5, 40, 41). Interestingly, our transcriptome sequencing (RNA-seq) data reveal that AtxA strongly represses the expression of BCAA transport and biosynthesis genes (15), and the regulation is possibly mediated by the AtxA-controlled small regulatory RNA XrrA (4).

Here, we describe the genomic arrangement of BCAA-related genes in B. anthracis (GenBank accession number AE017334). BCAA biosynthesis genes are clustered into two operons, while BrnQ-related BCAA transporter genes are present in multiple loci. We assess the function and specificity of putative BrnQ BCAA transporters and examine the relationships between BCAAs and AtxA. Finally, we test the virulence of mutants disrupted for BCAA transport and biosynthesis in a murine model for systemic infection. Our data indicate that both BCAA transport and synthesis are required for B. anthracis virulence, establishing a connection between central metabolism and pathogenesis in B. anthracis.

## RESULTS

### Growth of B. anthracis under toxin-inducing conditions with and without BCAAs.

Previous studies have shown that B. anthracis growth in various media is largely affected by isoleucine, leucine, and/or valine (33, 42, 43). We wanted to test the requirement for BCAAs when B. anthracis is cultured in a defined medium under conditions known to promote the synthesis of the anthrax toxin proteins. Ristroph medium (R medium) (10) was formulated based on the content of a Casamino Acids (CA) medium that was used previously to promote toxin synthesis (44, 45). R medium contains defined concentrations of glucose, salts, amino acids, vitamins, and nucleotides. Toxin synthesis is maximized when B. anthracis is cultured in CA (undefined) or R (defined) medium containing 0.8% bicarbonate and incubated at 37°C with shaking in a 5% CO_2_ atmosphere (10, 11, 38, 45).

**FIG 1:**
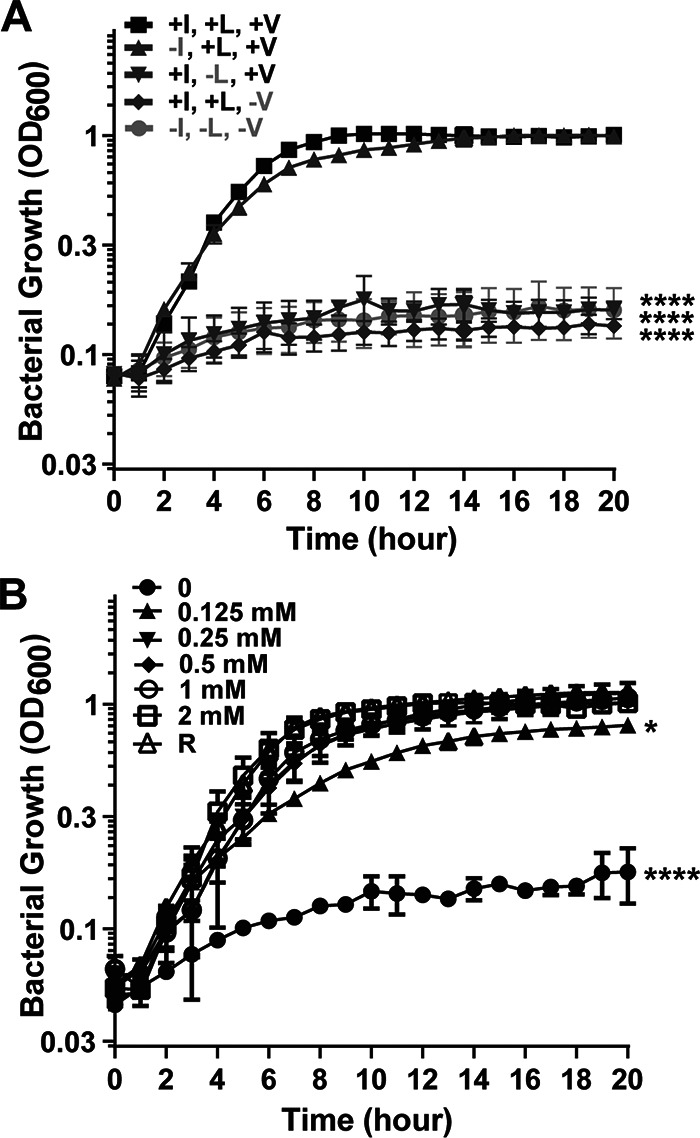
Requirement of BCAAs for B. anthracis ANR-1 growth under toxin-inducing conditions. (A) Growth in R medium (1.75 mM isoleucine, 1.5 mM leucine, and 1.35 mM valine) and R medium missing one or more BCAAs, as indicated. (B) Growth in R medium with altered concentrations of BCAAs. Each of the three BCAAs was present at the concentrations indicated. Data are the means from three biological replicates, with error bars representing standard deviations. Data were compared with growth in R medium and analyzed using one-way analysis of variance (ANOVA) followed by Dunnett’s multiple-comparison analysis. Asterisks indicate *P* values (*, *P* < 0.05; ****, *P* < 0.0001).

The BCAA content of R medium is 1.75 mM isoleucine, 1.50 mM leucine, and 1.35 mM valine (11). As shown in Fig. 1A, B. anthracis grown in R medium under toxin-inducing conditions exhibited a doubling time of approximately 1 h and a final optical density at 600 nm (OD_600_) of about 1. The absence of all three BCAAs resulted in minimal growth of the bacterium during 20 h of incubation under these conditions. Furthermore, culture in R medium in the absence of individual BCAAs revealed that leucine and valine are critical for B. anthracis growth. The poor growth in the absence of these amino acids suggests that B. anthracis relies on the transport, rather than the biosynthesis, of leucine and valine for optimal growth. In contrast, the absence of isoleucine had a relatively small but reproducible adverse effect on growth, indicating that isoleucine biosynthesis is sufficient for growth under these conditions, but the transport of the amino acid offers a small growth advantage.

To explore the BCAA concentrations required for growth under toxin-inducing culture conditions, we cultured B. anthracis in R medium and R medium with altered concentrations of BCAAs (Fig. 1B). Again, the absence of all BCAAs resulted in poor growth. Growth was restored when all BCAAs were present at 0.125 mM, and BCAA concentrations of at least 0.25 mM resulted in growth rates and yields comparable to those obtained in R medium. The importance of BCAAs for the optimal growth of B. anthracis under toxin-inducing conditions prompted us to examine the genome for predicted BCAA biosynthesis and transport genes.

### Expression of BCAA biosynthesis genes of B. anthracis.

**FIG 2:**
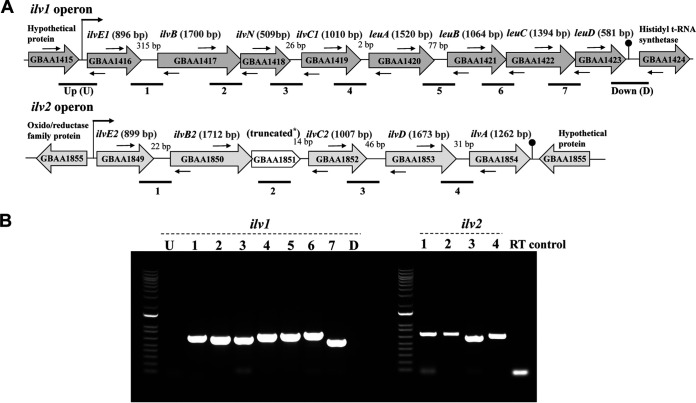
Organization and expression of *ilv* loci in B. anthracis. (A) Schematic representation of the *ilv* loci. Two operons designated operon *ilv1* and operon *ilv2* are shown. Genes are indicated as open arrows with the corresponding annotations (GenBank accession number AE017334). Gene sizes (base pairs) are shown in parentheses. truncated* is the truncation of a gene predicted to encode the small subunit of acetolactate synthase III. The sizes of intergenic spaces (base pairs) are indicated. Predicted transcription start and termination sites are denoted by bent arrows and lollipops, respectively. Thin horizontal arrows represent primer pairs for PCR. Small thin arrows above and below the genes indicate the approximate positions of primers used for RT-PCR. Horizontal lines below the operons correspond to the RT-PCR products shown in panel B. (B) Cotranscription of *ilv* loci. Ethidium bromide-stained agarose gels show the RT-PCR products obtained using the primers shown in panel A. Lane designations correspond to the anticipated products shown in panel A. The RT control reaction mixture contained RNA as a template. Primers directly upstream (U) and downstream (D) of the *ilv1* operon were used as negative controls for cotranscription with genes flanking the *ilv1* operon in the same DNA strand.

The interconnected pathways for isoleucine, leucine, and valine biosynthesis are highly conserved in bacteria (18, 23). Our analysis of the annotated B. anthracis genome revealed two loci, which we designated *ilv1* and *ilv2*, that together contain all genes required for the biosynthesis of BCAAs (12, 13). Some BCAA biosynthesis genes have alleles in both loci, while others exist in a single copy, as depicted in Fig. 2A. Both loci carry copies of *ilvE*, *ilvB*, and *ilvC*. The enzymes encoded by these genes are required for the synthesis of all three BCAAs. In contrast, *ilvD*, encoding another enzyme needed for the synthesis of all BCAAs, is present in a single copy on *ilv2*. The *ilv2* locus also contains *ilvA*, encoding an enzyme specific for isoleucine biosynthesis. Genes specific for leucine biosynthesis, *leuA*, *leuB*, *leuC*, and *leuD*, are clustered together in the *ilv1* locus. Notably, while the sequences of B. anthracis BCAA biosynthesis genes are indicative of proteins that are highly homologous to the well-characterized enzymes of B. subtilis, the gene arrangement in B. anthracis differs from that found in B. subtilis. The BCAA biosynthesis genes of B. subtilis are found in three loci, the *ilv-leu* operon, the *ilvA* gene, and the *ilvD* gene, and all of the genes are present in single copies (46).

We performed reverse transcription (RT)-PCR experiments to assess the expression of the BCAA biosynthesis genes. Using RNA from cultures grown in R medium with 0.25 mM BCAAs and primers corresponding to intergenic and flanking regions, we detected RT-PCR products indicating the cotranscription of all genes within each locus (Fig. 2B). Thus, *ilv1* and *ilv2* represent operons. The transcription of the BCAA biosynthesis operons was also detected in RNA from cultures grown in complete R medium (data not shown).

### Predicted BCAA transport genes of B. anthracis.

Despite the expression of BCAA biosynthesis genes, our experiments showed apparent BCAA auxotrophy during culture in defined media (Fig. 1). The importance of exogenous BCAAs for culture of B. anthracis prompted us to examine the B. anthracis genome for predicted BCAA transporter genes. Our bioinformatic analyses revealed that B. anthracis carries an unusually large number of predicted BCAA transporter genes compared to other Gram-positive bacteria for which BCAA transport has been studied. B. anthracis harbors six genes, *brnQ1* (GBAA_690), *brnQ2* (GBAA_0802), *brnQ3* (GBAA_1459), *brnQ4* (GBAA_2063), *brnQ5* (GBAA_3142), and *brnQ6* (GBAA_4790), predicted to encode BCAA transport system carrier II proteins. These proteins are LIVCS (leucine, isoleucine, and valine cationic symporter) family proteins, which function by a Na^+^ or H^+^ symport mechanism (47). They typically contain 12 transmembrane helices and are members of the major facilitator superfamily (MFS) (18, 48). The genome also contains five genes, GBAA_1931, GBAA_1933, GBAA_1934, GBAA_1935, and GBAA_1936, predicted to be ABC transporters for BCAAs as well as another gene, GBAA_0818, that is similar to *bcaP*, an amino acid permease gene that is found in many Gram-positive bacteria (16, 29, 31, 49). In contrast, the pathogens S. aureus and S. pneumoniae harbor four and one known or predicted BCAA transporters, respectively (16, 21, 22). The nonpathogen B. subtilis harbors three BCAA transporter genes (29). C. glutamicum, a bacterium used for the industrial synthesis of amino acids, carries only one BCAA transport gene (30), and L. lactis, used in the production of fermented milk products, harbors two BCAA transport genes (31). The relatively large number of apparent BCAA transporter genes of B. anthracis supports the importance of BCAA transport in this bacterium.

### Characterization of *brnQ*-null mutants.

**FIG 3:**
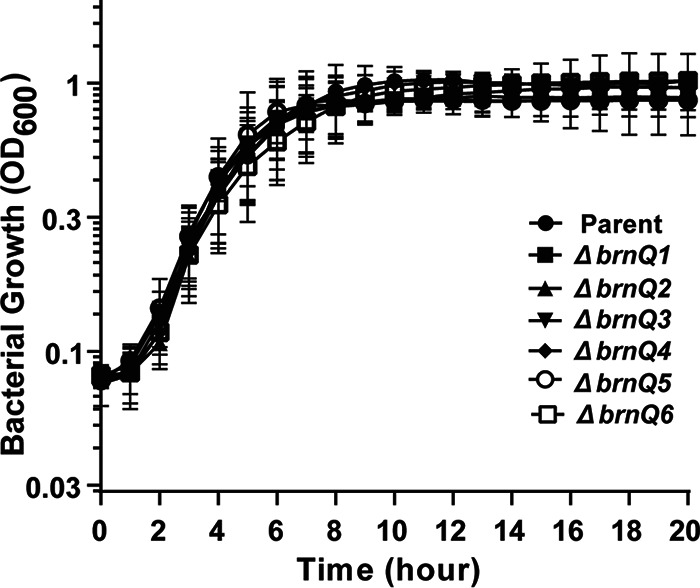
Growth of single *brnQ* transporter-null mutants in R medium. Each data point represents the average from three independent experiments ± the standard deviation. Data were analyzed using one-way ANOVA followed by Dunnett’s multiple-comparison test and compared with the data for the parent strain.

The presence of multiple genes predicted to encode BCAA transporters coupled with previous reports of controlled expression of the B. anthracis *brnQ* genes in response to regulators and signals associated with virulence (4, 15, 33, 34) spurred us to investigate BrnQ function. All six BrnQ paralogs are similar in length (BrnQ1, 433 amino acids [aa]; BrnQ2, 438 aa; BrnQ3, 451 aa; BrnQ4, 445 aa; BrnQ5, 450 aa; BrnQ6, 441 aa), and they share at least 42% sequence identity and 62% sequence similarity. Each protein is predicted to have 12 transmembrane domains, except for BrnQ4, which has 11 such domains (TMPred program [https://bio.tools/TMPred]) (50). We created individual *brnQ*-null mutants and compared their growth to that of the parent strain when cultured in R medium under toxin-inducing conditions. All six mutants showed growth rates comparable to that of the parent, revealing that no single *brnQ* gene is essential for B. anthracis growth under culture conditions in which BCAA transport is required (Fig. 3).

We performed BCAA transport assays to assess the ability of the parent strain, individual *brnQ*-null mutants, and complemented mutants to take up radiolabeled isoleucine, leucine, and valine. For complementation of the null mutations, the appropriate genes were cloned under the control of a xylose-inducible promoter, and expression was induced with 1% xylose. Although the assays were performed using native *brnQ* genes in the complemented mutants, we verified the induction system by cloning recombinant *brnQ* genes carrying a FLAG tag at the 3′ terminus, and protein expression was confirmed by immunoblotting with anti-FLAG antibody (data not shown).

**FIG 4:**
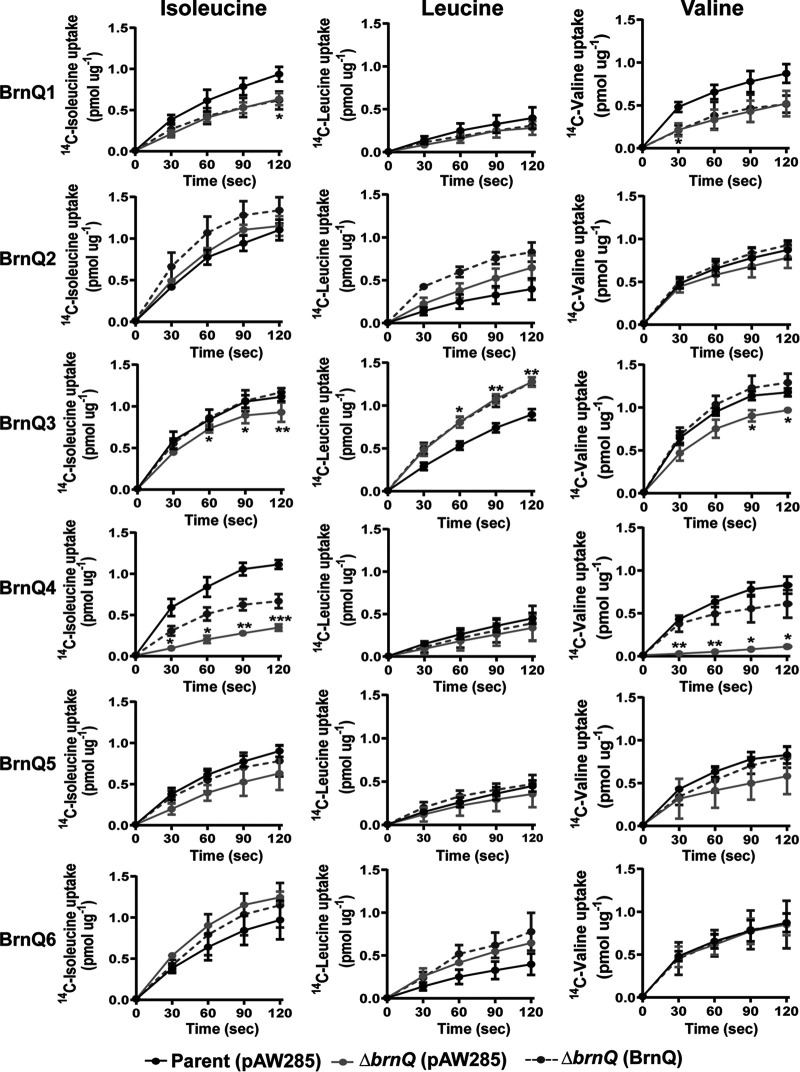
BCAA uptake by single *brnQ*-null mutants. The uptake of ^14^C-labeled isoleucine, ^14^C-labeled leucine, and ^14^C-labeled valine was assessed for the parent strain containing empty vector pAW285, an individual *brnQ* mutant with pAW285, and an individual *brnQ* mutant complemented with the corresponding gene. Data represent the means from three biological replicates ± standard deviations. Data were analyzed using two-way ANOVA with repeated measures followed by Bonferroni’s multiple-comparison analysis. Comparisons of the parent and single-deletion mutants are shown with asterisks representing *P* values (*, *P* < 0.05; **, *P* < 0.01; ***, *P* < 0.001).

The results of the BCAA transport assays are shown in Fig. 4. A strong phenotype was associated with *brnQ4*. Isoleucine and valine uptake were reduced substantially in the *brnQ4*-null mutant compared to the parent strain, and the phenotype was partially complemented by the expression of *brnQ4* in *trans*. The deletion of *brnQ4* did not affect leucine uptake. These data suggest that BrnQ4 is a major transporter for isoleucine and valine under these growth conditions. The deletion of *brnQ3* also resulted in a statistically significant decrease in isoleucine and valine uptake, and the expression of *brnQ3* in *trans* restored the mutant to the parent phenotype. Interestingly, the *brnQ3*-null mutant exhibited increased leucine uptake, a phenotype that was not complemented by *brnQ3* in *trans*. The deletion of *brnQ1* resulted in a small but statistically significant decrease in isoleucine and valine uptake; however, complementation by the expression of *brnQ1* in *trans* was not achieved. Individual deletions of the other *brnQ* genes did not result in statistically significant changes in BCAA uptake.

Overall, our assessment of growth and BCAA uptake by the single *brnQ*-null mutants suggests that multiple BrnQ transporters with various levels of functional redundancy and specificity are involved in BCAA uptake.

### Specificity of BrnQ transporters.

**FIG 5:**
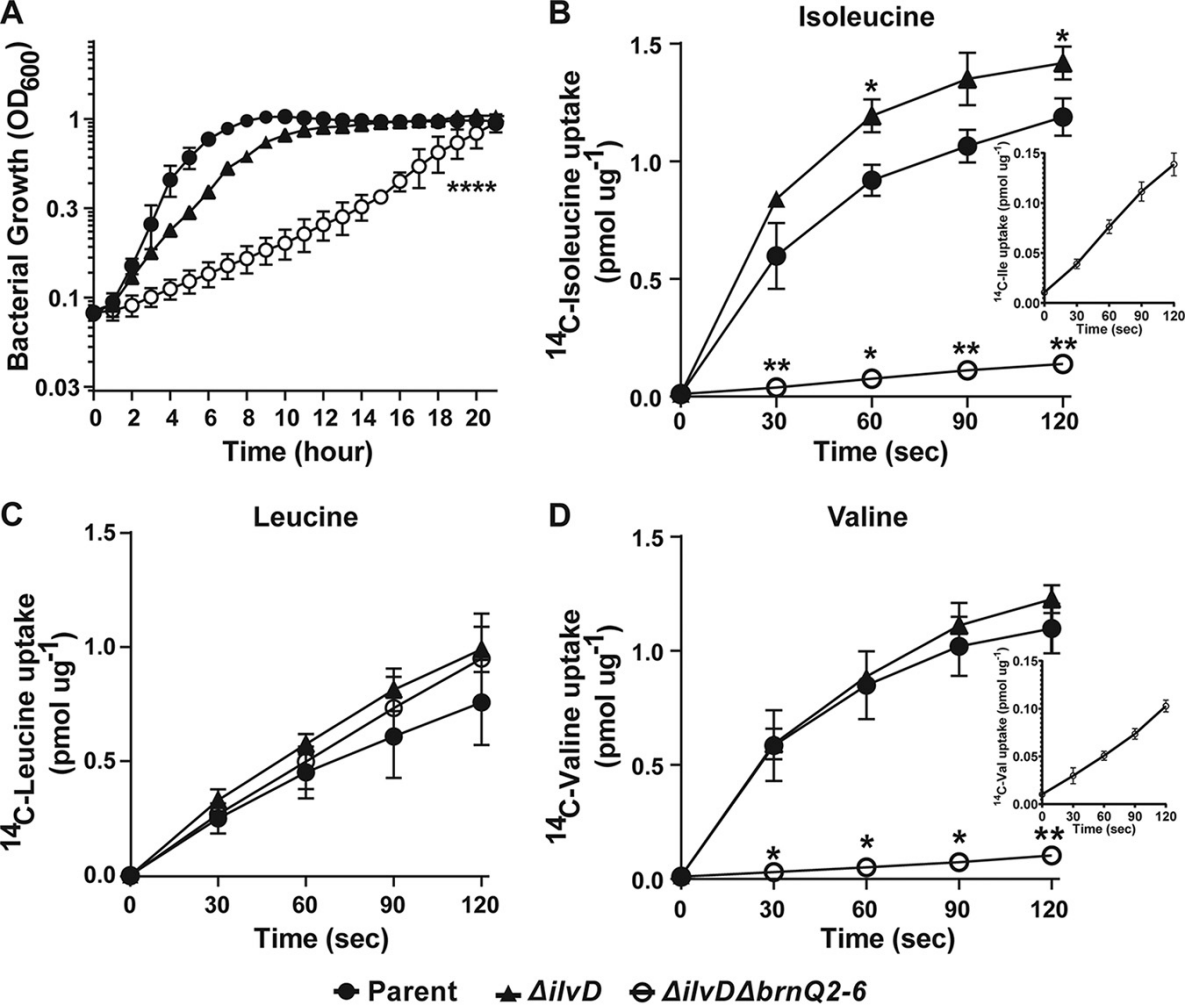
Growth and BCAA uptake by the Δ*ilvD* and Δ*ilvD*Δ*brnQ2–6* mutants. (A) Growth of the Δ*ilvD* and Δ*ilvD*Δ*brnQ2–6* mutants in R medium. Data are presented as the means from three independent experiments. Growth was compared to the respective growth of the parent. Error bars represent standard deviations. Data were analyzed using one-way ANOVA followed by Dunnett’s multiple-comparison analysis. Asterisks indicate *P* values (****, *P* < 0.0001). (B to D) BCAA uptake. The uptake of ^14^C-labeled isoleucine (B), ^14^C-labeled leucine (C), and ^14^C-labeled valine (D) was assessed for the parent, Δ*ilvD*, and Δ*ilvD*Δ*brnQ2–6* strains. The uptake of isoleucine and valine by the Δ*ilvD*Δ*brnQ2–6* mutant is shown in the insets of panels B and D, respectively. Data are the means from three biological replicates ± standard deviations. Data were analyzed using two-way ANOVA with repeated measures followed by Bonferroni’s multiple-comparison analysis. Comparisons of the parent with the Δ*ilvD* mutant and of the parent with the Δ*ilvD*Δ*brnQ2–6* mutant were assessed and are shown with asterisks indicating *P* values (*, *P* < 0.05; **, *P* < 0.01).

To explore the specificity of BrnQ transporters, we individually expressed *brnQ* genes in *trans* in a mutant deleted for multiple *brnQ* genes that was also deficient for BCAA biosynthesis. We reasoned that the assessment of transporter activity might be enhanced in a mutant unable to synthesize BCAAs. To construct the mutant, we first deleted *ilvD*. The *ilvD* gene encodes dihydroxy acid dehydratase, an enzyme essential for the biosynthesis of all three BCAAs. The growth rate of the *ilvD*-null mutant in R medium was reduced compared to that of the parent strain, but after 12 h, the densities of the two cultures were comparable (Fig. 5A). This result agrees with our above-described data (Fig. 1 and Fig. 2B) suggesting that while BCAA transport is sufficient to achieve optimal growth, BCAA biosynthesis is still active in media containing BCAAs.

Multiple attempts to create an *ilvD*-null mutant deleted for all 6 *brnQ* genes were unsuccessful, suggesting that the presence of at least one *brnQ* gene is essential for BCAA transport. The successive deletion of *brnQ2*, *brnQ3*, *brnQ4*, *brnQ5*, and *brnQ6* in the *ilvD*-null background resulted in a mutant, Δ*ilvD*Δ*brnQ2–6*, that exhibited a severe growth defect in R medium (Fig. 5A), likely due to significantly reduced BCAA transport. BCAA uptake by the Δ*ilvD*Δ*brnQ2–6* mutant was compared to those of the parent strain and the *ilvD*-null mutant (Fig. 5B to D). As expected, BCAA uptake was enhanced in the *ilvD* mutant compared to the parent, with a statistically significant increase in isoleucine uptake and small but reproducible increases in leucine and valine uptake. The Δ*ilvD*Δ*brnQ2–6* mutant showed greatly reduced isoleucine and valine uptake (Fig. 5B and D), suggesting that one or more of the deleted *brnQ* genes encode major transporters of these BCAAs and that the reduced growth rate of the Δ*ilvD*Δ*brnQ2–6* mutant in R medium (Fig. 5A) is related to the poor uptake of isoleucine and valine by less active transporters. The Δ*ilvD*Δ*brnQ2–6* mutant was unaffected for leucine uptake (Fig. 5C), indicating that *brnQ1* and/or unidentified transporters are associated with leucine transport.

**FIG 6:**
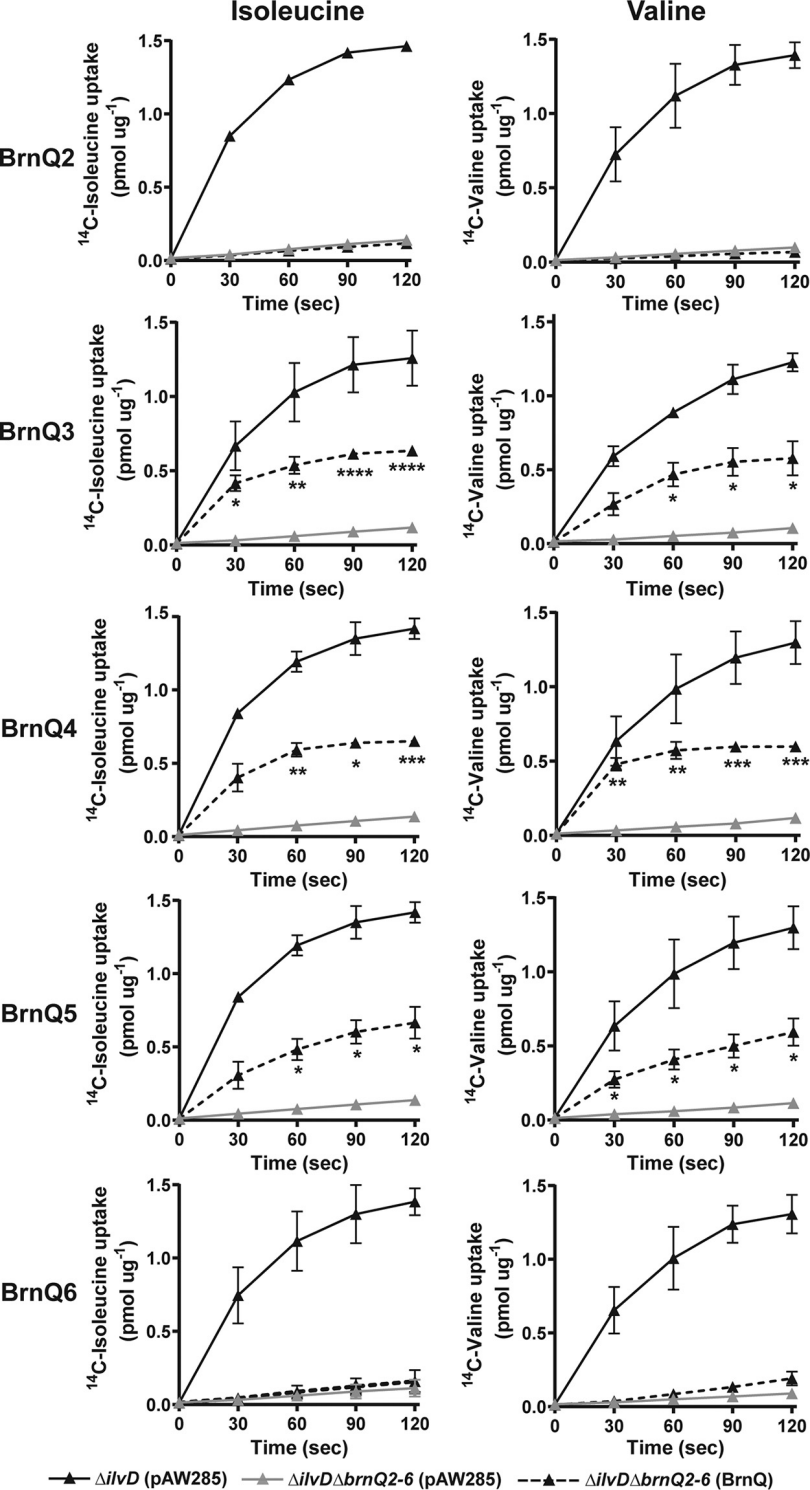
Isoleucine and valine uptake associated with the expression of specific BrnQs. The uptake of ^14^C-labeled isoleucine and valine was assessed for the Δ*ilvD* mutant containing the empty pAW285 vector, the Δ*ilvD*Δ*brnQ2–6* mutant containing the empty pAW285 vector, and the Δ*ilvD*Δ*brnQ2–6* strain complemented with individual *brnQ* genes. Values represent the means from three independent experiments ± standard deviations. Data were analyzed using two-way ANOVA with repeated measures followed by Bonferroni’s multiple-comparison analysis. Each complemented BrnQ^+^ mutant was compared to the Δ*ilvD*Δ*brnQ2–6* mutant and is shown with asterisks indicating *P* values (*, *P* < 0.05; **, *P* < 0.01; ***, *P* < 0.001; ****, *P* < 0.0001).

Next, we tested for complementation of the Δ*ilvD*Δ*brnQ2–6* mutant phenotype by individual *brnQ* genes. We assessed isoleucine and valine uptake associated with the expression of *brnQ2*, *brnQ3*, *brnQ4*, *brnQ5*, and *brnQ6* in *trans*. The expression of *brnQ3*, *brnQ4*, and *brnQ5* partially restored isoleucine and valine uptake by the Δ*ilvD*Δ*brnQ2–6* mutant, while the expression of *brnQ2* and *brnQ6* did not complement the uptake deficiency of the Δ*ilvD*Δ*brnQ2–6* mutant (Fig. 6). Taken together, our results indicate that BrnQ3, BrnQ4, and BrnQ5 can act as isoleucine and valine transporters.

**FIG 7:**
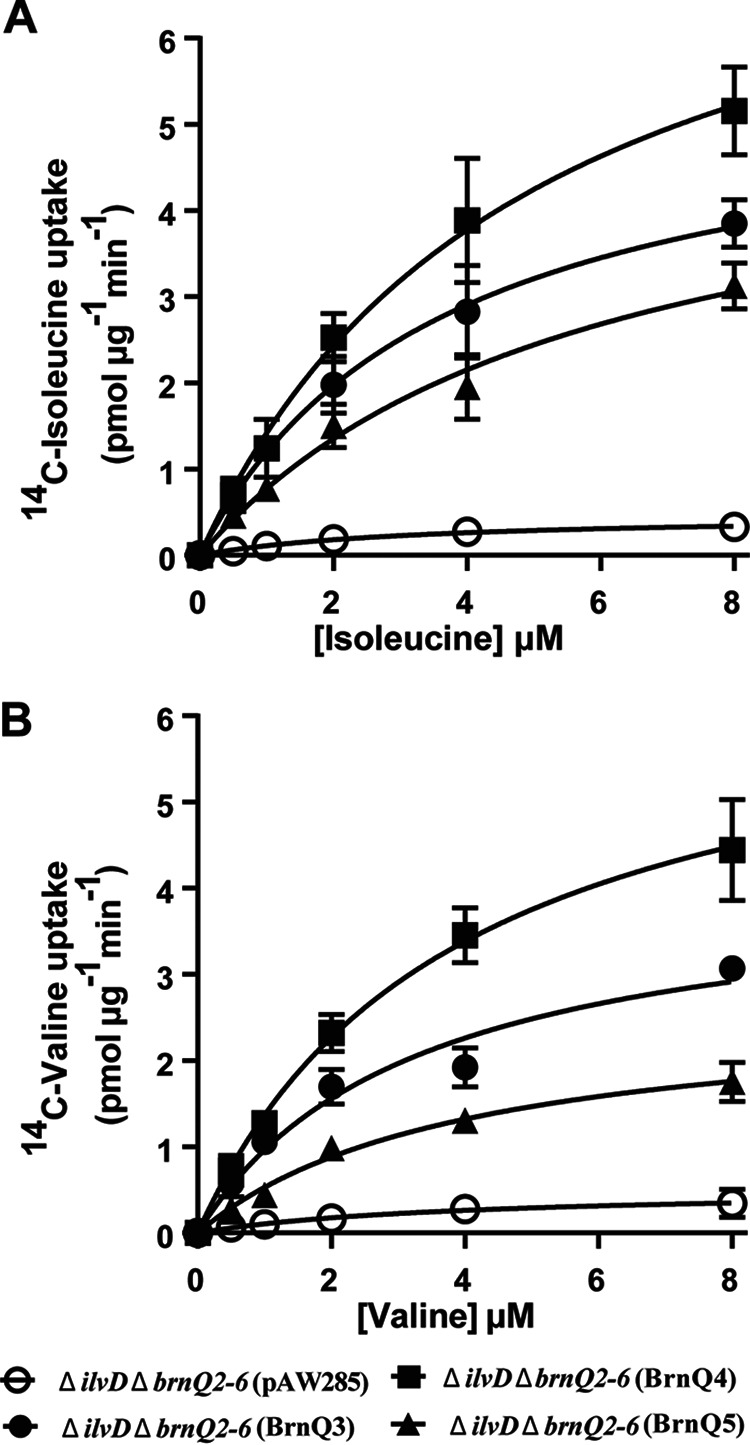
Kinetics of isoleucine and valine uptake associated with BrnQ3, BrnQ4, and BrnQ5. Isoleucine (A) and valine (B) uptake kinetics were assessed for the Δ*ilvD*Δ*brnQ2–6* strain with empty vector pAW285, the Δ*ilvD*Δ*brnQ2–6* strain with BrnQ3, the Δ*ilvD*Δ*brnQ2–6* strain with BrnQ4, and the Δ*ilvD*Δ*brnQ2–6* strain with BrnQ5. The data shown represent the means from three independent experiments. Error bars represent standard deviations.

To compare the activities of BrnQ3, BrnQ4, and BrnQ5, we measured the initial velocities of BCAA uptake with increasing concentrations of isoleucine and valine by the Δ*ilvD*Δ*brnQ2–6* mutant expressing individual *brnQ3*, *brnQ4*, or *brnQ5* genes (Fig. 7). The apparent *K_m_* and *V*_max_ values associated with each transporter are shown in Table 1. The data indicate that the three transporters have comparable affinities for isoleucine and valine under our experimental conditions. The highest initial velocity was associated with BrnQ4, suggesting that BrnQ4 is the major determinant for isoleucine and valine transport, which is in agreement with the strong phenotype of the *brnQ4*-null mutant in our BCAA uptake assays (Fig. 4). Altogether, our results establish roles for BrnQ3, BrnQ4, and BrnQ5 in BCAA transport by B. anthracis cultured under toxin-inducing conditions.

**TABLE 1:** Apparent *K_m_* and *V*_max_ values for the BrnQ3, BrnQ4, and BrnQ5 transporters

Transporter	Mean apparent *K_m_* (μM) ± SD		Mean apparent *V*_max_ (pmol μg^−1^ min^−1^) ± SD	
	Isoleucine	Valine	Isoleucine	Valine
BrnQ3	3.6 ± 1.6	3.3 ± 1.8	5.5 ± 1.2	4.1 ± 0.9
BrnQ4	4.9 ± 2.9	3.9 ± 1.6	8.5 ± 2.5	6.7 ± 1.3
BrnQ5	5.8 ± 3.7	4 ± 2.5	5.3 ± 2.3	2.6 ± 0.8

### Role of BCAA transport and synthesis in virulence.

**FIG 8:**
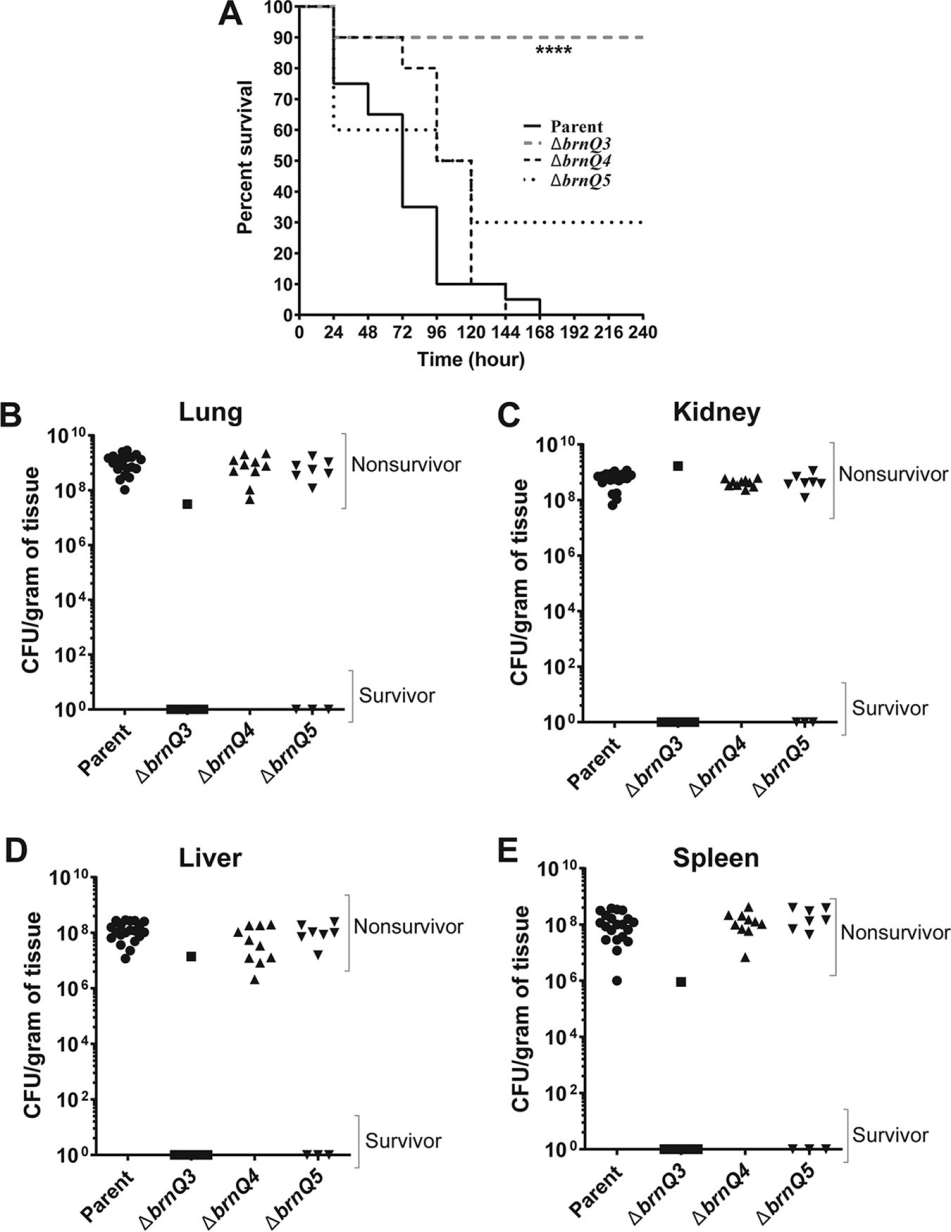
Virulence of BCAA transporter mutants. Seven- to eight-week-old female A/J mice were infected with ∼10^5^ CFU of the ANR-1 parent strain (*n* = 20), the Δ*brnQ3* mutant (*n* = 10), the Δ*brnQ4* mutant (*n* = 10), or the Δ*brnQ5* mutant (*n* = 10) via tail vein injection. Mice were monitored for 11 consecutive days. Organs were collected from dead and surviving mice, homogenized, and plated for the determination of CFU. (A) Kaplan-Meier survival curves for the parent, Δ*brnQ3* mutant, Δ*brnQ4* mutant, and Δ*brnQ5* mutant strains. Statistical significance was analyzed using the log rank (Mantel-Cox) test and compared with the parent. The *P* value is indicated by asterisks (****, *P* < 0.0001). (B to E) CFU in lungs (B), kidneys (C), livers (D), and spleens (E) from nonsurvivors and survivors were determined for the parent strain (circles), the Δ*brnQ3* mutant (squares), the Δ*brnQ4* mutant (triangles), and the Δ*brnQ5* mutant (inverted triangles). No detectable CFU were found in the organs of survivors. Individual data points are shown. Data for each mutant were compared to data for the parent. Mann-Whitney unpaired Student’s *t* test was used to determine significance.

To assess the significance of BrnQ3, BrnQ4, and BrnQ5 during B. anthracis infection, we tested *brnQ*-null mutants for virulence and tissue burden in a murine model for systemic anthrax. Groups of 7- to 8-week-old complement-deficient female A/J mice were infected intravenously with ∼10^5^ CFU of the parent strain or single *brnQ*-null mutants. Mice were monitored for up to 11 days. All parent strain-infected mice succumbed to anthrax disease within 8 days of infection (Fig. 8A). The time to death for mice infected with *brnQ4*- and *brnQ5*-null mutants was not significantly different from the time to death for mice infected with the parent strain. However, the *brnQ3*-null mutant was highly attenuated in this animal model. Nine of ten mice infected with this mutant survived infection and exhibited no signs of disease.

We collected the lungs, kidneys, livers, and spleens from surviving and diseased mice to determine the bacterial burdens in tissues. For all mice that succumbed to infection, comparable numbers of CFU were found in tissues infected with the parent strain and mutants (Fig. 8B to E). Approximately 10^9^ CFU per g of tissue was found in the lung and kidney, and approximately 10^8^ CFU per g of tissue was found in the liver and spleen. Thus, for diseased mice, the absence of a single isoleucine/valine transporter did not affect the numbers of the bacterium in various tissues. No detectable CFU were found in mice that survived infection.

Overall, these data show that BrnQ3 is required for full virulence even in the presence of other isoleucine/valine transporters, BrnQ4 and BrnQ5. The results indicate that despite the ability of BrnQ3, BrnQ4, and BrnQ5 to transport isoleucine and valine in cultured cells, these transporters do not have redundant activity during infection.

**FIG 9:**
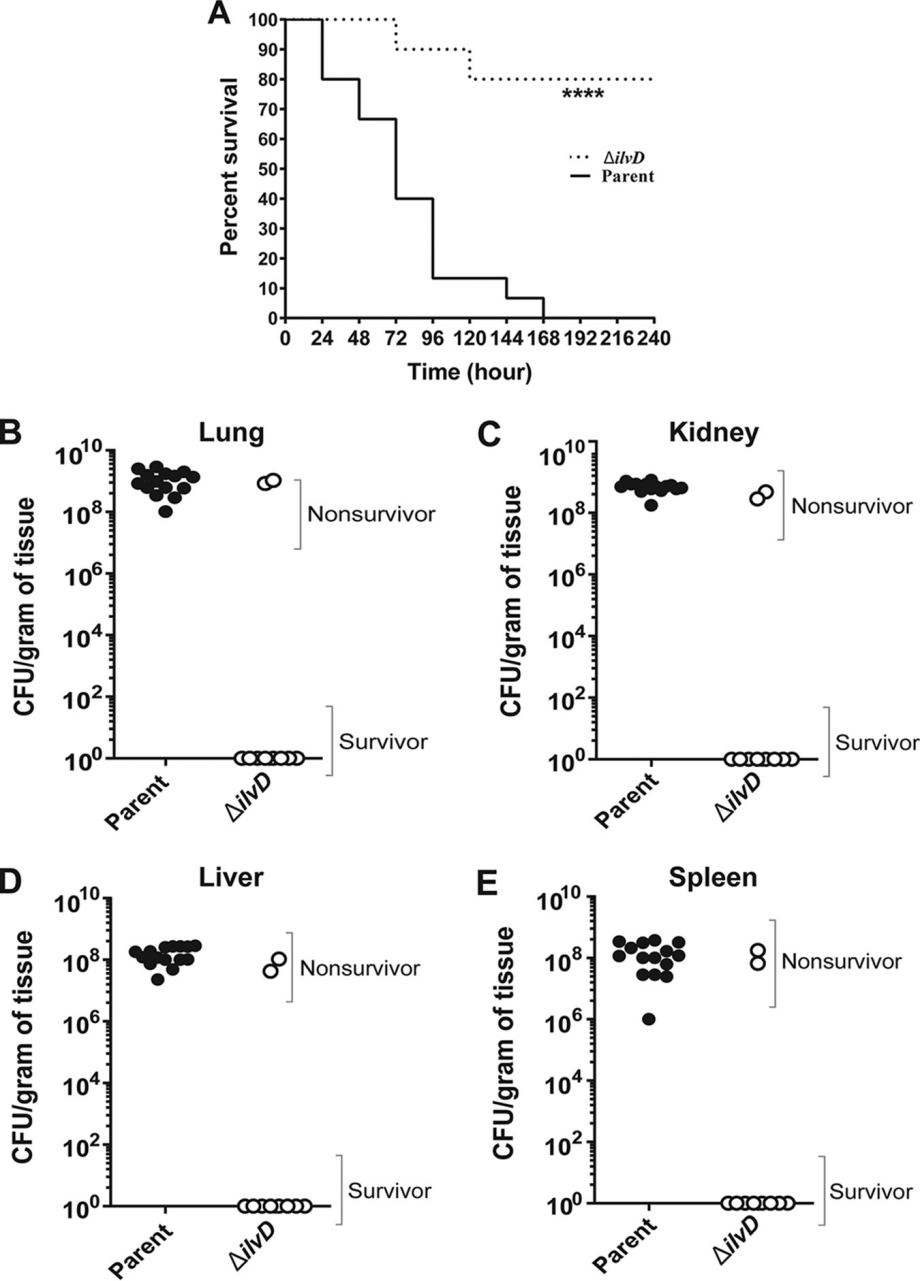
Virulence of a BCAA biosynthesis mutant. Mice were infected as described in the legend of Fig. 8 with the parent strain (*n* = 15) and the *ilvD*-null mutant (*n* = 10). (A) Kaplan-Meier survival curves for the parent strain and the Δ*ilvD* mutant. Statistical significance was analyzed by the log rank (Mantel-Cox) test, and the *P* value is denoted by asterisks (****, *P* < 0.0001). (B to E) CFU values in collected lungs (B), kidneys (C), livers (D), and spleens (E) of nonsurvivors and survivors for the parent (closed circles) and *ilvD*-null mutant (open circles) strains. The significance of the differences was analyzed by Mann-Whitney unpaired Student’s *t* test.

Our data showed that the deletion of *ilvD* has a minimal effect on B. anthracis growth in culture when all transporter genes are present. To determine if BCAA biosynthesis is important during infection, we also tested the *ilvD*-null mutant for virulence. Surprisingly, the *ilvD*-null mutant was attenuated (Fig. 9A). Eighty percent of the mice injected with the *ilvD*-null mutant did not exhibit symptoms, survived infection, and had no recoverable CFU in their tissues. For *ilvD*-null mutant-infected mice that succumbed to anthrax disease, the numbers of CFU in tissues from the lung, kidney, liver, and spleen were similar to those found in mice infected with the parent strain (Fig. 9B to E). These results suggest that while *ilvD* is not essential for growth in culture medium containing BCAAs, *ilvD* plays a role in virulence in the murine model.

### BCAA levels in mouse tissues.

Considering the attenuated virulence of a mutant deficient in BCAA biosynthesis and a mutant lacking the isoleucine/valine transporter BrnQ3, we sought to determine the relative abundance of BCAAs in mouse tissues. BCAA availability in mammalian tissues is largely undefined (18). Tissues from three uninfected mice were harvested, amino acids were extracted, and samples were analyzed by liquid chromatography (LC)-mass spectrometry (MS). The relative abundance of each BCAA was normalized by tissue weight (Table 2; see also Fig. S1 in the supplemental material). Of the four organs tested, the spleen contained the highest concentrations of all three BCAAs. For each organ, valine was the most abundant BCAA, while isoleucine was the least abundant BCAA. Overall, only ∼2- to 3-fold differences were noted between organs, suggesting somewhat comparable BCAA availabilities in these niches.

**TABLE 2:** BCAA levels in several mouse tissues

BCAA	BCAA level (nmol mg^−1^ tissue)			
	Lung	Liver	Spleen	Kidney
Isoleucine	0.08–0.13	0.13–0.18	0.19–0.23	0.12–0.14
Leucine	0.15–0.2	0.22–0.28	0.39–0.41	0.21–0.22
Valine	0.2–0.32	0.26–0.31	0.46–0.54	0.27–0.30

10.1128/mbio.03640-21.1 FIG S1 Graphical representation of BCAAs in mouse tissues. (A) Isoleucine; (B) leucine; (C) valine. One-way ANOVA followed by Tukey’s multiple-comparison test was used for data analysis. Each bar represents the mean from three biological replicates. Error bars represent standard deviations. Asterisks indicate *P* values (*, *P* < 0.05; **, *P* < 0.01; ***, *P* < 0.001; ****, *P* < 0.0001). Download FIG S1, TIF file, 0.9 MB. Copyright © 2022 Dutta et al. 2022 Dutta et al. https://creativecommons.org/licenses/by/4.0/ This content is distributed under the terms of the Creative Commons Attribution 4.0 International license.

### Influence of BCAAs on AtxA activity.

AtxA is a critical positive regulator of the anthrax toxin genes and the B. anthracis capsule biosynthesis operon (35, 37, 51). In our murine model for anthrax, death of the animal is highly dependent upon the synthesis of the anthrax toxin proteins, and an *atxA-*null mutant, which is toxin deficient, is avirulent (36, 38). Our more recent investigations show that in addition to the control of toxin and capsule genes, AtxA negatively affects the expression of BCAA transport- and synthesis-related genes (15), most likely via an indirect mechanism dependent upon a small regulatory RNA, XrrA (4). Transcriptional profiling data suggest that AtxA activates the transcription of XrrA (15), and XrrA represses the BCAA genes (4). To address the potential relationships between BCAAs and AtxA that could be associated with the attenuated virulence of the *ilvD*- and *brnQ3*-null mutants, we assessed *atxA* expression and AtxA activity during culture of B. anthracis with different levels of BCAAs.

**FIG 10:**
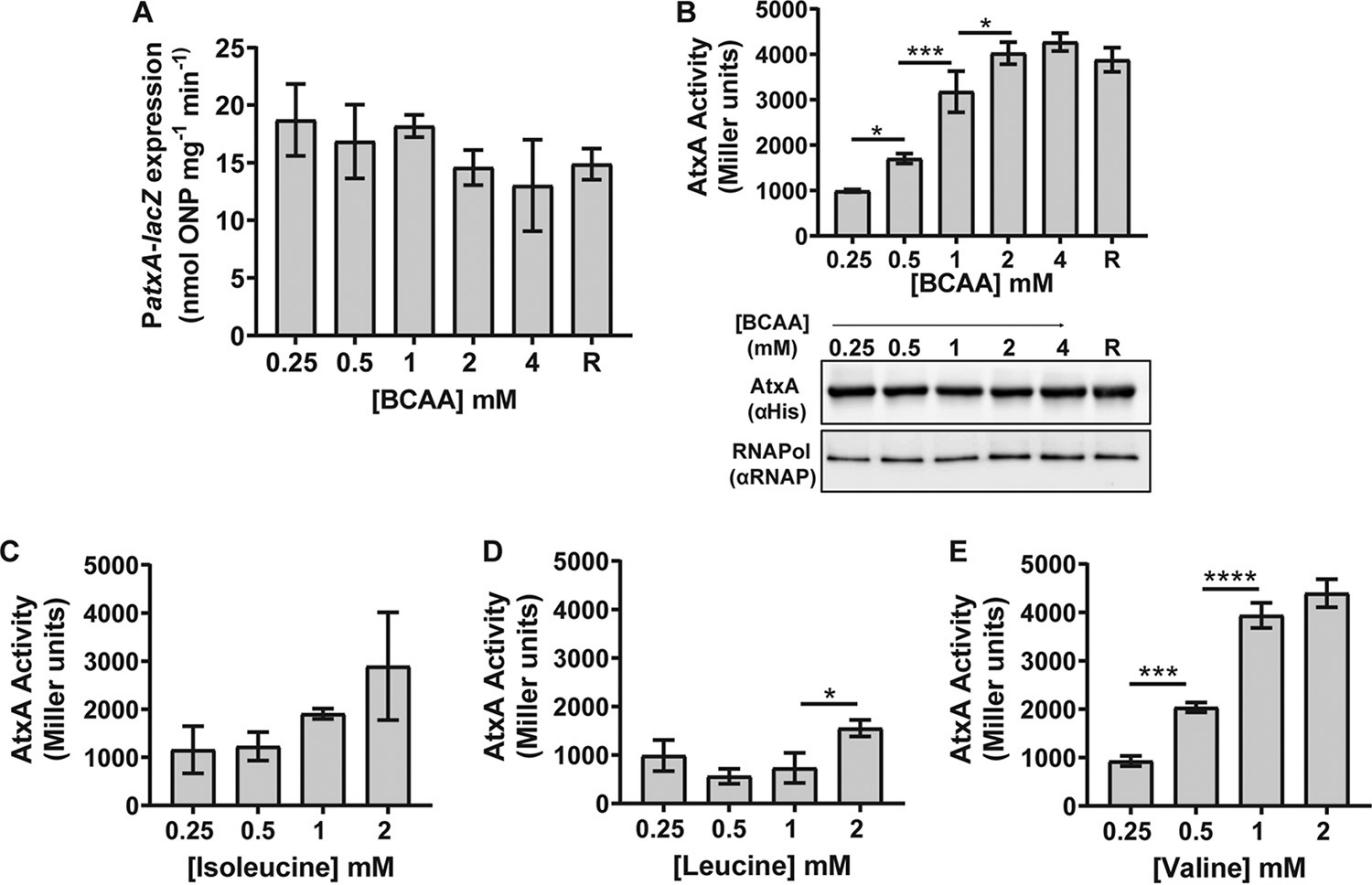
Effect of BCAAs on *atxA* promoter activity, AtxA function, and AtxA protein levels. (A) *atxA* promoter activity. The *atxA* promoter activity of a reporter strain carrying a P*atxA*-*lacZ* transcriptional fusion was measured using a β-galactosidase assay following growth with various BCAA concentrations. (B) AtxA protein activity *in vivo*. An *atxA*-null strain (UT376) carrying P*lef-lacZ* (a reporter for AtxA activity) and containing an IPTG-inducible His-tagged *atxA* allele was induced during growth in R medium with various BCAA concentrations. β-Galactosidase activity (top) and AtxA protein levels (bottom) were measured. Steady-state levels of AtxA and the RNA polymerase β-subunit were detected in cell lysates via immunoblotting using anti-His antibody and anti-RNA Pol β antibody, respectively. The experiment was performed three times, and a representative image is shown. (C to E) Effects of isoleucine (C), leucine (D), and valine (E) on AtxA activity. β-Galactosidase assays were performed as described above for panel B. The concentration of one BCAA (as indicated) was altered while keeping the concentrations of the other BCAAs constant at 0.25 mM to support optimal growth. Each bar represents the mean from three biological replicates. Error bars represent standard deviations. One-way ANOVA followed by Tukey’s multiple-comparison test was performed to analyze the data. Asterisks indicate *P* values (*, *P* < 0.05; ***, *P* < 0.001; ****, *P* < 0.0001).

To measure *atxA* promoter activity, we performed β-galactosidase assays using ANR-1(pUTE839), which carries an *atxA* promoter-*lacZ* fusion (Table 3). As shown in Fig. 10A, no significant differences in β-galactosidase activity were detected in cultures grown in R medium with BCAA concentrations ranging from 0.25 mM to 4 mM.

**TABLE 3:** B. anthracis strains and plasmids

Strain or plasmid	Relevant characteristic(s)	Reference
Strains		
ANR-1	B. anthracis parent strain; pXO1^+^ pXO2^−^	72
UT376	ANR-1 derivative; *atxA* null; *lef* promoter-*lacZ* fusion (P*lef*-*lacZ*) at the native *lef* locus	40
UT441	ANR-1 derivative; Δ*brnQ3*	This work
UT469	ANR-1 derivative; Δ*ilvD*	This work
UT472	ANR-1 derivative; Δ*brnQ2*	This work
UT475	ANR-1 derivative; Δ*brnQ5*	This work
UT476	ANR-1 derivative; Δ*brnQ6*	This work
UT478	ANR-1 derivative; Δ*brnQ4*	This work
UT481	ANR-1 derivative; Δ*brnQ1*	This work
UT482	ANR-1 derivative; Δ*ilvD* Δ*brnQ2* Δ*brnQ3* Δ*brnQ4* Δ*brnQ5* Δ*brnQ6* (Δ*ilvD*Δ*brnQ2–6*)	This work

Plasmids		
pHY304	Temp-sensitive vector used for deletion of the indicated loci by homologous recombination; Erm^r^	73
pAW285	Xylose-inducible expression vector, Cm^r^	70
pUTE839	pHT304-18z-derived *atxA* promoter-*lacZ* fusion vector containing the sequence from positions −770 to +99	38
pUTE991	pUTE657-derived expression vector for AtxA-His_6_ (6×His epitope on the C terminus of AtxA)	40
pUTE1165	pAW285-derived expression vector for BrnQ3-FLAG (FLAG tag on the C terminus of BrnQ3)	This work
pUTE1167	pAW285-derived expression vector for BrnQ6-FLAG (FLAG tag on the C terminus of BrnQ6)	This work
pUTE1191	pAW285-derived expression vector for BrnQ2	This work
pUTE1192	pAW285-derived expression vector for BrnQ3	This work
pUTE1193	pAW285-derived expression vector for BrnQ6	This work
pUTE1206	pAW285-derived expression vector for BrnQ1	This work
pUTE1207	pAW285-derived expression vector for BrnQ4	This work
pUTE1208	pAW285-derived expression vector for BrnQ5	This work
pUTE1209	pAW285-derived expression vector for BrnQ1-FLAG (FLAG tag on the C terminus of BrnQ1)	This work
pUTE1210	pAW285-derived expression vector for BrnQ2-FLAG (FLAG tag on the C terminus of BrnQ2)	This work
pUTE1211	pAW285-derived expression vector for BrnQ4-FLAG (FLAG tag on the C terminus of BrnQ4)	This work
pUTE1212	pAW285-derived expression vector for BrnQ5-FLAG (FLAG tag on the C terminus of BrnQ5)	This work

We quantified AtxA activity using reporter strain UT376, an *atxA*-null mutant harboring an isopropyl-β-d-thiogalactopyranoside (IPTG)-inducible His-tagged *atxA* allele in *trans*. This strain also contains a transcriptional fusion in which the promoter of an AtxA-regulated gene, *lef*, is fused to a promoterless *lacZ*, at the native *lef* locus. As shown in Fig. 10B (top), BCAAs enhanced AtxA activity in a dose-dependent manner, with maximum activity at 2 mM BCAAs, which is comparable to the BCAA concentration in R medium. The results of Western blot experiments indicated that the AtxA protein levels were similar in cultures containing different BCAA concentrations (Fig. 10B, bottom).

We repeated the AtxA activity experiments, varying the level of either isoleucine, leucine, or valine while keeping the other two BCAAs at the minimum concentration required to support optimal growth (0.25 mM). Valine exhibited a statistically significant dose-dependent effect on AtxA activity (Fig. 10E), while the effects of isoleucine and leucine were variable (Fig. 10C and D). These data show that BCAAs, and specifically valine, increase AtxA activity in a dose-dependent manner, linking BCAAs with virulence factor expression.

## DISCUSSION

Our studies of the significance of BCAA biosynthesis and transport by B. anthracis were prompted by data indicating that BCAA-related gene expression was altered by host-related signals and the virulence regulator AtxA. We were also intrigued by the unusually large number of *brnQ* genes associated with BCAA transport in B. anthracis compared to most other firmicutes. We have determined that although B. anthracis can synthesize BCAAs, BCAA transport is required for optimal growth in culture and for virulence in a mouse model for anthrax. Moreover, we have obtained evidence for some functional redundancy among the multiple BrnQ transporters and identified major transporters of isoleucine and valine.

Other pathogenic *Bacillus* species, including B. cereus and B. thuringiensis, also possess an abundance of *brnQ* genes. B. anthracis, B. cereus, and B. thuringiensis have a large degree of chromosome gene synteny and share multiple physiological processes, but the three species have vastly different pathogenic lifestyles (52). While B. anthracis is capable of causing lethal systemic infections in mammals, certain strains of B. cereus that produce emetic toxins and enterotoxins are causative agents of food poisoning and extraintestinal disease (53, 54), and some strains of B. thuringiensis are insect pathogens (55). Studies of BCAA synthesis and metabolism have not been reported for B. cereus and B. thuringiensis, but the presence of at least six putative BrnQ BCAA transporters in each species is intriguing given their differences in host interactions.

The apparent BCAA auxotrophy of B. anthracis despite the presence of BCAA biosynthesis genes is puzzling but not unprecedented among firmicutes. L. monocytogenes and S. aureus also carry genes that produce BCAA biosynthesis enzymes but exhibit auxotrophic ambiguity (24, 27). The molecular mechanism(s) for this phenotype is not clear. In these species, BCAA biosynthesis is subject to multiple layers of control, including attenuation and CodY-mediated repression (27, 56). CodY, first identified in B. subtilis as a repressor of the dipeptide transport (*dpp*) operon (57), uses BCAAs and GTP as effectors to enhance DNA-binding activity at consensus sequences. CodY homologs in many Gram-positive bacteria have been reported to control a wide range of genes, including some virulence factors.

In B. anthracis, putative CodY-binding sites appear upstream of *ilvE1* and *ilvB2* and also in the promoter regions of *brnQ2*, *brnQ4*, *brnQ6*, and the BCAA ABC transporter gene GBAA_1931 (58), but CodY-mediated regulation of these genes has not been reported. Interestingly, reports of CodY function in B. anthracis indicate an indirect role for CodY in toxin and capsule gene expression via AtxA. CodY does not affect the transcription or translation of *atxA*. Rather, the regulator controls AtxA protein stability by an unknown mechanism (59). Our experiments examining *atxA* promoter activity and AtxA protein levels in cultures containing increasing levels of BCAAs showed no change in AtxA expression or protein levels in response to BCAAs. Rather, our data revealed that BCAAs, most specifically valine, increase AtxA activity in a dose-dependent manner. Thus, BCAAs appear to be a signal for virulence gene expression in B. anthracis.

The mechanism for BCAA-enhanced AtxA activity is not known, but we surmise that it is CodY independent. van Schaik and coworkers (59) reported reduced AtxA protein levels in a *codY-*null mutant. We have been unable to construct a *codY-*null mutant in our parent strain. Yet our experiments demonstrating BCAA activation of AtxA, taken together with our previous reports showing negative regulation of BCAA-related genes by XrrA (4) and positive regulation of XrrA by AtxA (15), suggest a feedback loop in which AtxA controls its own activity by regulating BCAA levels in cells. Although we did not find appreciable differences in the BCAA contents of different mouse tissues, it is possible that niche-specific differences in BCAA availability could serve to modulate AtxA activity for optimal pathogenesis. Further investigations of the relationships between BCAAs and AtxA are needed to dissect the molecular mechanism for this apparent fine-tuning of AtxA activity.

Our experiments examining growth and BCAA transport by mutants deleted for individual *brnQ* genes revealed that no single BrnQ transporter is essential for B. anthracis growth under our culture conditions. These findings are consistent with functional redundancy among the transporters. While none of the single *brnQ*-null mutants displayed growth defects under our culture conditions, some of the mutants were affected for BCAA transport, and complementation of mutants by inducing the expression of the corresponding gene in *trans* resulted in elevated transport of some BCAAs. For example, the deletion of *brnQ4* significantly decreased isoleucine and valine uptake, suggesting that BrnQ4 plays a predominant role in the transport of these BCAAs. A smaller reduction in isoleucine and valine uptake was observed for a *brnQ3*-null mutant. Interestingly, the uptake deficiency of the *brnQ3*-null mutant was fully restored by expressing *brnQ3* in *trans*, but the expression of *brnQ4* in *trans* in the *brnQ4*-null mutant resulted in only a partial restoration of isoleucine and valine transport.

We were unable to delete all *brnQ* genes in a single strain, indicating that at least one *brnQ* gene is essential for growth in culture. To test for specific function, we expressed individual *brnQ* genes in *trans* in a mutant deleted for *ilvD* and five of the six *brnQ* genes. We chose to create the Δ*ilvD*Δ*brnQ2–6* mutant harboring an intact *brnQ1* gene for these studies based on BCAA uptake by single mutants and previously reported transcriptomic analyses. The expression of *brnQ1* is not affected during growth in blood, and it is not controlled by AtxA (15, 34). The single *brnQ1*-null mutant showed a weak transport phenotype compared to other *brnQ*-null mutants (a small reduction in isoleucine and valine uptake), but the phenotype was not complemented by expressing *brnQ1* in *trans*. A similar phenotype was associated with the single *brnQ5*-null mutant, but reduced isoleucine and valine uptake was recovered when *brnQ5* was expressed in *trans*. All other *brnQ* genes were of interest because they were highly regulated and/or single null mutants exhibited relatively strong transport phenotypes that could be complemented. The Δ*ilvD*Δ*brnQ2–6* mutant was viable under our culture conditions but exhibited a significantly reduced growth rate, affirming the importance of BrnQ transporters for B. anthracis physiology. Furthermore, the mutant showed a significant deficiency for isoleucine and valine uptake, suggesting that BrnQ1 does not play a major role in the transport of these BCAAs. Data from our BCAA transport assays employing the Δ*ilvD*Δ*brnQ2–6* mutant expressing individual BrnQ transporters revealed significant functional overlap for BrnQ3, BrnQ4, and BrnQ5, and strains expressing each of these transporters did not show significant differences in the kinetics of isoleucine and valine transport.

Assessment of leucine uptake by BrnQ3, BrnQ4, and BrnQ5 was not feasible because the Δ*ilvD*Δ*brnQ2–6* mutant is capable of transporting leucine under our experimental conditions. Leucine transport may be associated with BrnQ1 or other uncharacterized non-BrnQ-type transporters. The B. anthracis genome sequence indicates a locus, GBAA_1931, GBAA_1933, GBAA_1934, GBAA_1935, and GBAA_1936, predicted to encode a BCAA-associated ABC transporter. Major leucine transporters have not been identified in other *Bacillus* species. However, a putative BCAA permease of B. subtilis, YvbW, has been reported to be regulated by a leucine-specific T-box mechanism and is a candidate for a leucine transporter (29, 60). We did not find a homolog of YvbW in the B. anthracis genome. A leucine-specific T box is present upstream of *brnQ6* (60), yet in our experiments, leucine uptake is unaffected in a *brnQ6*-null mutant.

Assignment of BCAA-specific function to BrnQ proteins is challenging not only because of functional redundancy but also because the regulation of the transporter genes is likely very complex. Experiments employing null mutants and strains overexpressing *brnQ* genes in other bacteria have resulted in surprising phenotypes. For example, in S. aureus, the deletion of *brnQ2*, encoding an isoleucine-specific transporter, affects the expression of *brnQ1*, which encodes the major transporter for isoleucine, leucine, and valine (21). The expression level of *brnQ1* is over 40-fold higher in a *brnQ2*-null mutant than in the parent strain. The overexpression of *brnQ1* results in the elevated transport of isoleucine, leucine, and valine (21). Our B. anthracis data reveal the possibility of similar relationships in which one transporter affects the expression or function of another. The *brnQ3*-null mutant exhibited increased leucine uptake, a phenotype that was not complemented by the expression of *brnQ3* from a xylose-inducible promoter in *trans*. It is possible that the deletion of *brnQ3* elevated the expression of a leucine transporter. In addition, the growth of the Δ*ilvD*Δ*brnQ2–6* mutant in the absence of BCAAs, although slow compared to that of the parent, and the low rate of transport of isoleucine and valine by the mutant indicate the presence of other additional transporters of these BCAAs. In this mutant, BrnQ1 or a non-*brnQ*-type transporter may compensate for the absence of BrnQ3, BrnQ4, and BrnQ5.

The expression and function of BCAA transporters in the context of the host may differ from expression and function during culture. In our mouse model for systemic anthrax, *brnQ3* was essential for infection, whereas the *brnQ4*- and *brnQ5*-null mutants were fully virulent. BrnQ3-dependent virulence suggests that access to isoleucine and/or valine is critical for growth during infection and that BrnQ3 serves as the major transporter of these BCAAs. It is possible, but less likely, that the absence of *brnQ3* alters the expression of other transporters that are important for virulence. The importance of BCAA transport during infection has been reported for S. aureus and S. pneumoniae (16, 21, 22). In S. aureus, both BrnQ1 and BcaP are required for full virulence in a murine nasal-colonization and hematogenous-spread infection model (16, 21). The S. pneumoniae branched-chain amino acid ABC transporter LivJHMGF is essential for virulence in a murine pneumonia model but is not necessary for nasopharyngeal colonization (22).

The attenuated phenotype of the *brnQ3*-null mutant suggests a limited availability of BCAAs in mouse tissues, resulting in growth restriction and/or reduced AtxA activity. Our LC-MS data revealed 0.08 to 0.23 nmol isoleucine mg^−1^ tissue, 0.15 to 0.41 nmol leucine mg^−1^ tissue, and 0.20 to 0.54 nmol valine mg^−1^ tissue. It is difficult to compare the concentrations of BCAAs in solid tissues to those present in liquid, but it is worth noting that BCAA levels in human blood range from 0.02 mM to 0.27 mM, and BCAA concentrations in porcine plasma are between 0.08 mM and 0.20 mM (18, 61, 62). Notably, in human blood and porcine plasma, as was true for our murine tissue samples, isoleucine is the least prevalent BCAA. Considering that the minimum concentration of BCAAs required for robust B. anthracis growth in culture is 0.25 mM, it is not unreasonable to consider these host niches as BCAA limited.

The restricted availability of BCAAs in the animal is further supported by the inability of the *ilvD*-null mutant to establish infection in our animal model. The deletion of BCAA biosynthesis genes in other bacteria harboring apparent BCAA transporter genes, including L. monocytogenes and S. pneumoniae, also results in reduced virulence (20, 63). We note that while the manuscript was in review, Jelinski et al. reported work indicating that an *ilvD*-null mutant of B. anthracis strain 34F2 was not attenuated in a murine model using intranasal infection with spores (64). The bacterial strain and infection models differed from those employed in our study, and the basis for this conflicting result will be examined in future studies.

Taken together, our data show that both BCAA transport and synthesis are critical for B. anthracis virulence in a murine model for late-stage anthrax. Further investigations will be directed at determining the control mechanisms for BCAA-related genes and the potential roles of BCAAs as host-related signals relevant for pathogenesis.

## MATERIALS AND METHODS

### Strains and growth conditions.

Bacterial strains and plasmids used in this study are described in Table 3. B. anthracis strain ANR-1 (Ames nonreverting) was used as the parent strain. Escherichia coli TG1 was used for regular cloning and transformation. E. coli strains GM2163 and SCS110 (lacking *dam* and *dcm*) were used to isolate nonmethylated plasmid DNA for the electroporation of B. anthracis (65).

E. coli strains were cultured in Luria-Bertani (LB) broth (66) with shaking (150 rpm) or on LB agar plates at 37°C, with the exception of strains carrying the temperature-sensitive pHY304 vector, which were cultured at 30°C. LB agar was also used for plating B. anthracis. When appropriate, the following antibiotics were used: spectinomycin (50 μg mL^−1^ for E. coli and 100 μg mL^−1^ for B. anthracis), erythromycin (150 μg mL^−1^ for E. coli and 5 μg mL^−1^ for B. anthracis), and chloramphenicol (7.5 μg mL^−1^ for both E. coli and B. anthracis).

B. anthracis strains were precultured overnight at 30°C in 25 mL of brain heart infusion (BHI) medium (Becton, Dickinson and Company, Franklin Lakes, NJ, USA) in a 250-mL flask with shaking (180 rpm). For each strain, 5 mL of a culture grown overnight was centrifuged at 4,000 rpm for 5 min, washed twice with phosphate-buffered saline (PBS), and then resuspended in PBS. The resuspended cells were transferred to a starting OD_600_ of 0.08 in 25 mL of Casamino Acids medium supplemented 0.8% sodium bicarbonate (CA-CO_3_) (44, 49) or in modified Ristroph medium (R medium) (10, 11). Unless indicated otherwise, the BCAA concentrations in R medium were the same as those reported previously (11): 1.75 mM isoleucine, 1.5 mM leucine, and 1.35 mM valine. Cultures were further incubated at 37°C with shaking in the presence of 5% atmospheric CO_2_ (toxin-inducing conditions) (67) until the desired time point or OD_600_ was reached. Growth curves for B. anthracis strains were performed in a 24-well sterile covered microplate using a BioTek Synergy HT plate reader. The plate was incubated at 37°C in 5% atmospheric CO_2_ with continuous orbital shaking (365 cpm), and the OD_600_ was determined at 1-h intervals.

### Recombinant DNA techniques.

B. anthracis and E. coli were cultured overnight in 2 mL of LB broth. Genomic DNA was obtained from the B. anthracis cultures using the Ultraclean microbial DNA isolation kit (Mo Bio Laboratories, Inc., Carlsbad, CA). Plasmid DNA isolation from E. coli was performed using the QIAprep Spin miniprep kit (Qiagen, Inc., Valencia, CA, USA). Oligonucleotides were purchased from Sigma-Aldrich (St. Louis, MO, USA) or Integrated DNA Technologies, Inc. (Coralville, IA, USA). For DNA cloning, Phusion HF DNA polymerase, high-fidelity restriction enzymes, and T4 DNA ligase were purchased from either New England BioLabs (NEB) (Ipswich, MA, USA) or Thermo Scientific (Waltham, MA, USA). The QIAquick gel extraction kit and the QIAquick PCR purification kit (Qiagen, Inc., Valencia, CA, USA) were used for PCR product cleanup. All procedures were performed according to the manufacturer’s protocol. Colonies of potential recombinant mutants were screened using colony PCR with 2.0× *Taq* Red master mix (Genesee Scientific, USA). For colony PCR, a small quantity of cells was added to the PCR master mix, and the suspension was heated at 95°C for 15 min prior to PCR. DNA sequences were confirmed by sequencing.

### Recombinant strain construction.

Markerless gene deletions in B. anthracis were made by homologous recombination as described previously (5). Briefly, DNA fragments corresponding to sequences approximately 1 kb upstream and 1 kb downstream of the target genes were obtained using PCR with the corresponding oligonucleotide pairs (see Table S1 in the supplemental material). Overlapping PCR was performed to obtain ∼2-kb fragments representing the ligated flanking regions (68). Fragment preparations digested with the appropriate restriction enzymes were ligated into plasmid pHY304, which contains a heat-sensitive origin of replication and an erythromycin resistance cassette. Ligation mixtures were first transformed into E. coli TG1 (Table 3). Recombinant plasmids purified from TG1 were subsequently transformed into an E. coli strain lacking *dam* and *dcm* to obtain plasmid DNA for the electroporation of B. anthracis. B. anthracis strains were electroporated using a method described previously (5). Electroporants were passaged and plated onto LB agar plates (master plates). Colonies from master plates were replica plated onto LB agar and LB agar containing erythromycin to identify erythromycin-sensitive colonies. Sensitivity to erythromycin was indicative of the loss of the pHY304 construct with a potential double-crossover event resulting in the recombination of the cloned DNA into the chromosome. Gene deletions were confirmed using PCR and sequencing. For the creation of the Δ*ilvD*Δ*brnQ2–6* mutant, the *ilvD*, *brnQ2*, *brnQ3*, *brnQ4*, *brnQ5*, and *brnQ6* genes were deleted sequentially in multiple experiments using distinct pHY304-derived constructs. Newly constructed strains were stored as spores using a previously described protocol (69).

10.1128/mbio.03640-21.2 TABLE S1 Oligonucleotides used in this study. Download Table S1, PDF file, 1.7 MB. Copyright © 2022 Dutta et al. 2022 Dutta et al. https://creativecommons.org/licenses/by/4.0/ This content is distributed under the terms of the Creative Commons Attribution 4.0 International license.

### Expression of transporter proteins.

DNA corresponding to the sequences of the B. anthracis *brnQ1*, *brnQ2*, *brnQ3*, *brnQ4*, *brnQ5*, and *brnQ6* open reading frames was amplified from ANR-1 genomic DNA using PCR with the appropriate oligonucleotides shown in Table S1. To facilitate the detection of proteins encoded by these genes, amplification products carrying a sequence encoding a FLAG tag fused to the 3′ end of the gene were also generated. The purified PCR products were digested with the SalI and BamHI restriction enzymes and finally ligated into the shuttle vector pAW285 such that expression was dependent on the xylose-inducible promoter (70). As described above, recombinant plasmids were first generated in E. coli TG1 (Table 3) and subsequently transformed into an E. coli strain lacking *dam* and *dcm* to obtain unmethylated plasmid DNA for electroporation into B. anthracis. For the expression of the cloned genes, B. anthracis cultures were grown in CA-CO_3_ to an OD_600_ of ∼0.3 and induced with 1% xylose. After incubation for 3 h, cells were collected, and cell lysates were prepared as described previously (5). The production of FLAG-tagged transporter proteins was assessed by Western blotting using anti-FLAG antibody (GenScript, Piscataway, NJ, USA) as described previously (5).

### BCAA uptake assay.

Cells from cultures induced for transporter gene expression were collected using a Nalgene rapid-flow sterile vacuum filter unit (0.2-μm pore size) (Thermo Fisher, Rockford, IL, USA). Cells were washed twice with PBS, resuspended in R medium lacking BCAAs to an OD_600_ of 5.0, and kept on ice until use. A 100-μL aliquot of prepared cells was stored at −20°C for measuring the protein content. The transport assay protocol was adapted from previously published protocols (21, 32). Immediately before the assay, cells were incubated for 15 min at 37°C. The ^14^C-labeled amino acid of interest (Perkin-Elmer, MA, USA) was added to the cells to a final concentration of 1 μM with stirring. At time intervals of 0, 30, 60, 90, and 120 s, 100-μL aliquots were removed, added to 4 mL of ice-cold 0.1 M LiCl_2_, and filtered rapidly through Whatman GF/F, 25-mm glass microfiber filters (GE Healthcare, Chicago, IL, USA) using a 1225 sampling manifold filtration unit (EMD Millipore, Darmstadt, Germany) attached to a vacuum pump. The filters were further washed with 4 mL of ice-cold 0.1 M LiCl_2_. The washed filters were dried for 30 min under a heat lamp and placed into a 20-mL borosilicate glass scintillation vial containing 5 mL of the Ultima Gold F liquid scintillation cocktail (Perkin-Elmer, MA, USA). The ^14^C radioactivity retained on the filters was quantified using an LS6500 scintillation system (Beckman Coulter, Inc., CA, USA). The protein contents of 100-μL cell samples were measured using the Bio-Rad protein assay kit. Uptake rates were calculated from the retained activity and total added activity and expressed in picomoles of amino acid per microgram of protein.

For kinetic measurements, cells were prepared as described above and then incubated for 20 s with 0.5 μM, 1 μM, 2 μM, 4 μM, or 8 μM the radiolabeled substrate. Reactions were stopped by adding 4 mL of 0.1 mM ice-cold LiCl_2_, and cells were collected immediately using filtration. The initial velocity of uptake for each substrate was plotted using GraphPad Prism software version 9. Experiments were performed in triplicate, and *K_m_* and *V*_max_ values were determined using nonlinear kinetics with GraphPad Prism software version 9.

### Measurement of AtxA activity, AtxA protein levels, and *atxA* transcription.

To quantify *in vivo* AtxA activity, we used the markerless *atxA*-null B. anthracis reporter strain UT376 that harbors the AtxA-regulated transcriptional fusion P*lef*-*lacZ* and contains an IPTG-inducible hexahistidine-tagged *atxA* allele (pUTE991) (Table 3). Cultures were grown in R medium with various BCAA concentrations as indicated for 3 h and induced with 30 μM IPTG. After incubation for 4 h, the OD_600_ was determined. One-milliliter samples were used for β-galactosidase assays, and 4-mL aliquots were preserved to quantify the AtxA protein using immunoblotting. β-Galactosidase assays were performed as described previously (40), and results were expressed in Miller units. Western blotting was performed as described previously (5). His-tagged AtxA was detected using anti-His antibody (GenScript USA, Inc., Piscataway, NJ, USA). RNA polymerase (RNAP) subunit β (Pol β) was used as a loading control and detected using anti-RNAP antibody (Thermo Fisher, Rockford, IL, USA). Densitometry was performed using ImageJ software (71), and AtxA protein levels were normalized to the RNA polymerase β protein.

To assess *atxA* promoter activity, we used a B. anthracis reporter strain containing a pHT304-18z-derived *atxA* promoter-*lacZ* fusion (pUTE839) (38) (Table 3). Following induction with IPTG, 1-mL samples were collected for β-galactosidase assays. Additional 1-mL samples were used for total protein determination using the Bio-Rad protein assay kit. β-Galactosidase assays were performed as described previously (38). Promoter activity was expressed as ortho nitro phenol (ONP) per minute per milligram.

### Virulence studies.

Animal protocols were reviewed and approved by The University of Texas Health Science Center Institutional Animal Care and Use Committee and performed using accepted veterinary standards. Female 7- to 8-week-old A/J mice were purchased from The Jackson Laboratory (Bar Harbor, ME) and maintained in a pathogen-free environment. Mice were housed 5 per cage and allowed a period of 72 h to acclimate to their surroundings prior to use in experiments.

Infections and determinations of CFU in tissue were performed as described previously (4). Briefly, mice were sedated with 0.1 mg mL^−1^ acepromazine administered intraperitoneally. Sedated mice were infected with 100 μL of a vegetative cell suspension (∼10^5^ CFU) via tail vein injection. Mice were monitored four times daily for 11 days. Animals died naturally or were sacrificed upon the appearance of disease symptoms. The lungs, kidneys, livers, and spleens of deceased or sacrificed mice were aseptically excised, weighed, and placed individually into 1 mL of sterile Dulbecco’s PBS (DPBS) containing zirconia/silica beads with a diameter of 2.3 mm (BioSpec Products, Inc., Bartlesville, OK, USA). Organs were homogenized by bead beating for 1 min and then placed on ice. Tissue lysates were plated onto LB agar and incubated overnight at 37°C for CFU determination. Kaplan-Meier survival curves and other statistical analyses were performed with GraphPad Prism version 9.

### RNA isolation.

B. anthracis cultures were grown in R medium or R medium with 0.25 mM BCAAs for 5 h. Total RNA was extracted from 10 mL of the culture using the NucleoSpin RNA kit (Macherey-Nagel, Inc., PA, USA) as described by the manufacturer. RNA samples were quantified using a NanoDrop ND-1000 spectrophotometer. Isolated RNA was treated with RNase-free DNase (Qiagen, Inc., Valencia, CA, USA) for 2 h at room temperature to remove any genomic DNA contamination. RNA was then purified and concentrated using the RNA Clean and Concentrator kit (Zymo Research, Irvine, CA, USA). The removal of DNA contamination was verified by PCR using purified RNA as the template.

### Reverse transcription-PCR.

Nonquantitative reverse transcription (RT)-PCR was used to test for cotranscription of the *ilv* genes. Purified RNA was used for first-strand cDNA synthesis employing the Superscript III reverse transcriptase kit (Invitrogen, Carlsbad, CA, USA) and random hexamers. Synthesized cDNA was purified using a QIAquick PCR purification kit (Qiagen, Inc., Valencia, CA, USA), and the final cDNA concentration was determined using a NanoDrop spectrophotometer. Purified cDNA (45 ng) was used as the template with the primers shown in Table S1 to amplify transcripts extending across adjacent genes. The PCR products were examined in a 1.2% agarose gel after ethidium bromide staining.

### Branched-chain amino acid measurement in mouse tissues.

Lungs, livers, spleens, and kidneys were harvested from each of three uninfected 7- to 8-week-old female A/J mice, placed into 15-mL centrifuge tubes, and snap-chilled in liquid nitrogen. BCAA analysis was performed by the MD Anderson Metabolomics Core Facility (Houston, TX). A small portion of each tissue sample was weighed and transferred to a 2-mL Precellys tube containing ceramic beads (Bertin, France). Tissue samples (20 to 30 mg) were crushed in liquid nitrogen and homogenized using a Precellys tissue homogenizer (Bertin, France). Amino acids were extracted using 0.5 mL of ice-cold 90:10 (vol/vol) methanol-water containing 0.1% formic acid. Extracts were centrifuged at 17,000 × *g* for 5 min at 4°C, and the supernatants were transferred to clean tubes, followed by evaporation to dryness under nitrogen. Samples were reconstituted in 0.1% formic acid in 90:10 (vol/vol) acetonitrile-water and then injected for analysis by liquid chromatography (LC)-MS. LC mobile phase A (MPA) (weak) was acetonitrile containing 1% formic acid, and mobile phase B (MPB) (strong) was water containing 50 mM ammonium formate. A Thermo Vanquish LC system included an Imtakt Intrada amino acid column (3-μm particle size, 150 by 2.1 mm) with the column compartment kept at 30°C. The autosampler tray was chilled to 4°C. The mobile phase flow rate was 300 μL min^−1^, and the gradient elution program was as follows: 15% MPB from 0 to 5 min, 15 to 30% MPB from 5 to 20 min, 30 to 95% MPB from 20 to 30 min, 95% MPB from 30 to 40 min, 95 to 15% MPB from 40 to 41 min, and 15% MPB from 41 to 50 min. The total run time was 50 min. Data were acquired using a Thermo Orbitrap Fusion Tribrid mass spectrometer under electrospray ionization (ESI) positive-ionization mode at a resolution of 240,000. Raw data files were imported to Thermo Trace Finder software for final analysis. The relative abundance of each metabolite was normalized by tissue weight.
