# Supplementary figures and images for: BrnQ-Type Branched-Chain Amino Acid Transporters Influence Bacillus anthracis Growth and Virulence
Source: mBio. 2022 Jan 25;13(1):e03640-21. doi: 10.1128/mbio.03640-21 (PMC8787487; doi:10.1128/mbio.03640-21)

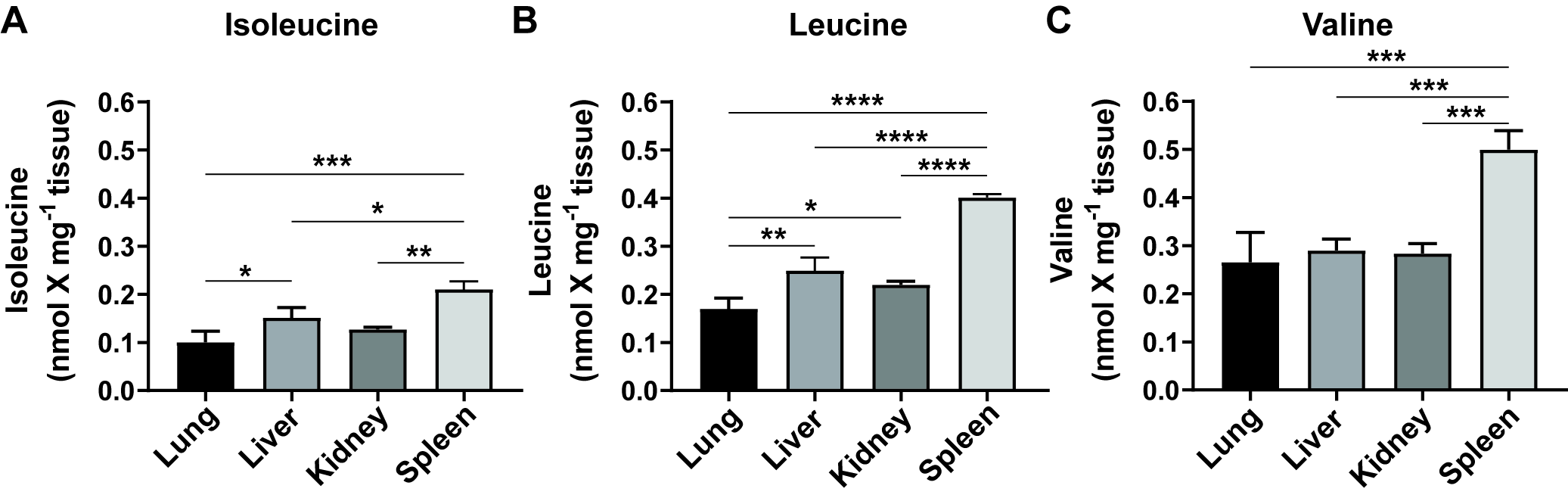

Supplement: FIG S1 [file mbio.03640-21-sf001.tif]
