# Supplementary material for: BrnQ-Type Branched-Chain Amino Acid Transporters Influence Bacillus anthracis Growth and Virulence
Source: mBio. 2022 Jan 25;13(1):e03640-21. doi: 10.1128/mbio.03640-21 (PMC8787487; doi:10.1128/mbio.03640-21)
Supplement: TABLE S1 [file mbio.03640-21-st001.pdf]

**Table S1: Oligonucleotides used in this study.**

| Name | Sequence (5' to 3') | Description |
|---|---|---|
| Oligonucleotides for gene deletions | | |
| SD180 | ACGCGTCGACGAACTACCAGCTTGTTCAAAGCCAGCC | *brnQ1* markerless deletion, forward primer for upstream region with *Sal*I site |
| SD181 | GGAAAACACCTCTTACTTACATTTCTTTCTCCTTCTTTAATTTC | *brnQ1* markerless deletion, reverse primer for upstream region, overlapping primer |
| SD182 | AAGAAGGAGAAAGAAATGTAAGTAAGAGGTGTTTTCCTATTTCG | *brnQ1* markerless deletion, forward primer for downstream region, overlapping primer |
| SD183 | CGGGATCCGAAAACTGCATTCGCACTAAATATCGCCAAGC | *brnQ1* markerless deletion, reverse primer for downstream region with *BamH*I site |
| SD208 | CCGGTCTGTTAAGTATCCACCAATCATTG | Forward primer for *brnQ1* deletion mutant check |
| SD209 | GTTAATAGTCCAGCATCTACCCCTGC | Reverse primer for *brnQ1* deletion mutant check |
| SD1 | CCCAAGCTTTTCTTCTCCTATTAGTTCTTTTGAGCATCG | *brnQ2* markerless deletion, forward primer for upstream region with *Hind*III site |
| SD2 | AGGATTTTCTTTATATGTAAAGAAAAGCAGCAAGCATGAG | *brnQ2* markerless deletion, reverse primer for upstream region, overlapping primer |
| SD3 | GCTTGCTGCTTTTCTTTACATATAAAGAAAATCCTCCTACTGAGTGTTTTTGT | *brnQ2* markerless deletion, forward primer for downstream region, overlapping primer |
| SD4 | CCGGAATTCTACTAGTCGAACTAACGCTTTACTTAGACA | *brnQ2* markerless deletion, reverse primer for downstream region with *EcoR*I site |
| SD5 | GGAGTCTATTTAGATCATTAACAGTGAATCCG | Forward primer for *brnQ2* deletion mutant check |
| SD6 | GCAACCTATATACAAGCTAAAGGTGATCC | Reverse primer for *brnQ2* deletion mutant check |
| NB114 | CTTAGTCGACGGTTCAGAGTTAGCACTGCAATTAAGACCGGATATTATT | *brnQ3* markerless deletion, forward primer for upstream region with *Sal*I site |
| NB115 | GGAAAAACAATGGCATCCTAAAAAAACTCCCAAAAGGGAGTTTTTTAC | *brnQ3* markerless deletion, reverse primer for upstream region, overlapping primer |
| NB116 | TTAGGATGCCATTGTTTTTCCTCCTTTTTTACATTCATTTGTAAGAACAC | *brnQ3* markerless deletion, forward primer for downstream region, overlapping primer |
| NB117 | CTTACTGCAGATTGCAAGATTTATCCCCCCGAACAGG | *brnQ3* markerless deletion, reverse primer for downstream region with *Pst*I site |
| SD7 | CTAAATGTAAGTGACCGGACACAGGCG | Forward primer for *brnQ3* deletion mutant check |
| SD8 | CGAATAATACTCCCATCGCAAGCATTCC | Reverse primer for *brnQ3* deletion mutant check |
| SD186 | ACGCGTCGACGTTCAAGTTCAATTACAATAGCGGAAGCACTGGAGCAT | *brnQ4* markerless deletion, forward primer for upstream region with *Sal*I site |
| SD187 | AAAAGAAAGGCGCCTACATGCTTTTCATTTGCATAGCAC | *brnQ4* markerless deletion, reverse primer for upstream region, overlapping primer |
| SD188 | CAAATGAAAAGCATGTAGGCGCCTTTCTTTTTTTAAAGGGG | *brnQ4* markerless deletion, forward primer for downstream region, overlapping primer |
| SD189 | CGGGATCCGCTGATTGTTGGTAGCATTCTTATGACTGCAGAGC | *brnQ4* markerless deletion, reverse primer for downstream region with *BamH*I site |

| | | |
|---|---|---|
| SD190 | GAAGTCGAAACGATAGAGTAACATGCAAAAGCC | Forward primer for *brnQ4* deletion mutant check |
| SD191 | CATCACCTGCTTCAGGTACTCCTCCTAATAC | Reverse primer for *brnQ4* deletion mutant check |
| NB130 | CTTAGTCGACCACCCGTAAAAACCCCACTT | *brnQ5* markerless deletion, forward primer for upstream region with *Sal*I site |
| NB131 | GGAGTTGTTATGTAAATTATTTTTGTTTATCATACCATTATCGGGC | *brnQ5* markerless deletion, reverse primer for upstream region, overlapping primer |
| NB132 | AATAATTTACATAACAACTCCTCCGATTATACATTTG | *brnQ5* markerless deletion, forward primer for downstream region, overlapping primer |
| NB133 | CTTACTGCAGCAAATAGGATTTATCGGATGTGGAAACA | *brnQ5* markerless deletion, reverse primer for downstream region with *Pst*I site |
| SD9 | ACAGTAAAGACCAACCTACTAGATGAG | Forward primer for *brnQ5* deletion mutant check |
| SD10 | GTGTATATGTTTATAGAAGCCATGGCAG | Reverse primer for *brnQ5* deletion mutant check |
| NB118 | CTTAGTCGACGCATCAAAAGCAAAACGAATTGTGTAGTGG | *brnQ6* markerless deletion, forward primer for upstream region with *Sal*I site |
| NB119 | GAGGTTAGTAAAGATGATGAAATAGAGAAAAGAGGCTTTCTATAATAGAAAGTCTCTTT | *brnQ6* markerless deletion, reverse primer for upstream region, overlapping primer |
| NB120 | TTTCATCATTTCTACTAACCTCCTAATTTAATTTAACAAAATATTCTGAATTAAAATCCG | *brnQ6* markerless deletion, forward primer for downstream region, overlapping primer |
| NB121 | CTTACTGCAGCACAGCAGCTGAGCATATTGAAGGTAATATTATTGATTATA | *brnQ6* markerless deletion, reverse primer for downstream region with *Pst*I site |
| SD11 | CGTATGGCATTACAAGAAAAGCTAGCATACATACG | Forward primer for *brnQ6* deletion mutant check |
| SD12 | GAAAGTGGCGAGACATTACAGCTGAAGAATT | Reverse primer for *brnQ6* deletion mutant check |
| SD100 | AACTGCAGGGAGTGTATGGAGTATGAAAACATATTATGAGCAAGATGC | *ilvD* markerless deletion, forward primer for upstream region with *Pst*I site |
| SD101 | TCTTTTATATTGACTCACATCTTGTCAAATCCCCTCTTAC | *ilvD* markerless deletion, reverse primer for upstream region, overlapping primer |
| SD102 | GGGATTTGACAAGATGTGAGTCAATATAAAAGAGATAAGGATG | *ilvD* markerless deletion, forward primer for downstream region, overlapping primer |
| SD103 | CGGGATCCCTTTATCAAGAAATTCTCTTAACGCACCTGAACGTT | *ilvD* markerless deletion, reverse primer for downstream region with *BamH*I site |
| SD104 | ATACGGCAGAGTTCGGGGATTATGTAAC | Forward primer for *ilvD* deletion mutant check |
| SD105 | CGCTTGCACAAACAACACCGTTTT G | Reverse primer for *ilvD* deletion mutant check |
| M13 F | TGTAAAACGACGGCCAGT | Forward primer for clone checking, for pHY304 vector backbone |
| M13 R | GGAAACAGCTATGACCATG | Reverse primer for clone checking, for pHY304 vector backbone |
| Oligonucleotides for cloning | | |

| | | |
|---|---|---|
| SD196 | CGGGATCCATGAAATTGTTACAGAAAAAAGAAATTTTACTTATTAGTCTTATGTTATTC | Forward primer for *brnQ1* cloning with *BamH*I site |
| SD197 | ACGCGTCGACTTA**TTTATCATCATCATCTTTATAATC**CTTTCTTCTTATTGAAAACAAAACTAACGATAACACCAATAC | Reverse primer for *brnQ1* cloning with *Sal*I site, FLAG tagged |
| SD198 | ACGCGTCGACTTATTTCTTCTTATTGAAAACAAAACTAACGATAACACCAATAC | Reverse primer for *brnQ1* cloning with *Sal*I site |
| SD199 | GGGATTGTGATTGCAAATGCTCTTCG | Sequencing primer for *brnQ1* clone check |
| SD61 | CGGGATCCATGCGTACAACTTTAAAACCAGCGCAAATAC | Forward primer for *brnQ2* cloning with *BamH*I site |
| SD58 | ACGCGTCGACTTA**TTTATCATCATCATCTTTATAATC**TTTTGTAGAAACTTCGCCTTCAG | Reverse primer for *brnQ2* cloning with *Sal*I site, FLAG tagged |
| SD145 | ACGCGTCGACTTATTTTGTAGAAACTTCGCCTTCAGCTTCTG | Reverse primer for *brnQ2* cloning with *Sal*I site |
| SD46 | TTGCAACAGTATTAACAAATGTTTC | Sequencing forward primer for *brnQ2* clone check |
| SD45 | CGATTGAAAGATAAATAATGATAGCG | Sequencing reverse primer for *brnQ2* clone check |
| SD62 | CGGGATCCATGAACACTGTATCAAAAAAACATATTTTTTCA | Forward primer for *brnQ3* cloning with *BamH*I site |
| SD56 | ACGCGTCGACTTA**TTTATCATCATCATCTTTATAATC**GGATGCTTTTACTTCACGAGATT | Reverse primer for *brnQ3* cloning with *Sal*I site, FLAG tagged |
| SD146 | ACGCGTCGACTTAGGATGCTTTTACTTCACGAGATTC | Reverse primer for *brnQ3* cloning with *Sal*I site |
| SD48 | TATCCAATTGCAATCGTACTTGTTC | Sequencing forward primer for *brnQ3* clone check |
| SD47 | GACCAATGGCAATGTATACAACTAC | Sequencing reverse primer for *brnQ3* clone check |
| SD200 | CGGGATCCATGAAGGGACGTTTAAGGCCAGGTG | Forward primer for *brnQ4* cloning with *BamH*I site |
| SD201 | ACGCGTCGACCTA**TTTATCATCATCATCTTTATAATC**AGCACCTTTCTTTTGTAGTTCACTTGATTCATTTG | Reverse primer for *brnQ4* cloning with *Sal*I site, FLAG tagged |
| SD202 | ACGCGTCGACCTAAGCACCTTTCTTTTGTAGTTCACTTGATTCATTTG | Reverse primer for *brnQ4* cloning with *Sal*I site |
| SD203 | CGATGGATGCAATTAGTGCACTTGTATTTG | Sequencing primer for *brnQ4* clone check |
| SD204 | CGGGATCCATGTCAAATAAAGTTCCTACTTCTTTTATCATTATTATAGGATTGA | Forward primer for *brnQ5* cloning with *BamH*I site |
| SD205 | ACGCGTCGACTTA**TTTATCATCATCATCTTTATAATC**TTTATTTAATTGAGTTTCACTAGAACTTAAGGTATTTTTCTTCC | Reverse primer for *brnQ5* cloning with *Sal*I site, FLAG tagged |
| SD206 | ACGCGTCGACTTATTTATTTAATTGAGTTTCACTAGAACTTAAGGTATTTTTCTTCC | Reverse primer for *brnQ5* cloning with *Sal*I site |
| SD207 | CAACGACTATTGCTGCTACACTCTTAG | Sequencing primer for *brnQ5* clone check |

| | | |
|---|---|---|
| SD63 | CGGGATCCATGAAATCATCTTTAAAGTT TTCTGAGATGT | Forward primer for *brnQ6* cloning with *BamH*I site |
| SD60 | ACGCGTCGACCTA**TTTATCATCATCAT CTTTATAATC**TTTTTCTACAGCAACTTC TTGC | Reverse primer for *brnQ6* cloning with *Sal*I site, FLAG tagged |
| SD147 | ACGCGTCGACCTATTTTTCTACAGCAAC TTCTTGCTTT | Reverse primer for *brnQ6* cloning with *Sal*I site |
| SD52 | ATTTATGGTAGCAAACCTTGGG | Sequencing forward primer for *brnQ6* clone check |
| SD51 | CGCCTAAACATAAATAAATTGCAGT | Sequencing reverse primer for *brnQ6* clone check |
| pAW2 85FP | CAGGCTTTACACTTTATGCTTCC | Forward primer for clone checking, for pAW285 vector backbone |
| pAW2 85RP | AAACAACAAACTAATAGGTGATGTACT TAC | Reverse primer for clone checking, for pAW285 vector backbone |
| | | |
| Oligonucleotides for assessing *ilv1* and *ilv2* operons | | |
| SD338 | CGGACGTCCGTTTATTGAATACAATAG | Forward primer, checking for upstream of *ilv1* operon |
| SD341 | TCGGATCGTCTCAACAACAATATTC | Reverse primer, checking for upstream of *ilv1* operon |
| SD106 | GACAGTAGATGGTAGAACGATCGG | Forward primer for fragment 1 within *ilv1* locus |
| SD107 | CTGGATTGCCAGTAATTCTGGCATA | Reverse primer for fragment 1 within *ilv1* locus |
| SD108 | GTTCGCATAGATGATCCACTTCTTG | Forward primer for fragment 2 within *ilv1* locus |
| SD109 | TGCTACGCGTAAAGGCTGTTA | Reverse primer for fragment 2 within *ilv1* locus |
| SD110 | CGGTTTGAAAGAAATTGCAAGAACAG | Forward primer for fragment 3 within *ilv1* locus |
| SD111 | ATAAGAAAGTGCCTTTTCAGTCGC | Reverse primer for fragment 3 within *ilv1* locus |
| SD112 | GAGGTTGGATTGCAGAGCATAAAG | Forward primer for fragment 4 within *ilv1* locus |
| SD113 | TTCTGGATTCGTATACCCAACTGTATC | Reverse primer for fragment 4 within *ilv1* locus |
| SD114 | CAGGTTTTGGTGTTGCACAAGA | Forward primer for fragment 5 within *ilv1* locus |
| SD115 | CTTCCGCTTACTCGCTAATTGAAAG | Reverse primer for fragment 5 within *ilv1* locus |
| SD116 | GTGTGCAATTGAAGAAGCGATTTC | Forward primer for fragment 6 within *ilv1* locus |
| SD117 | GCTACACCGTACTTTGAAAGGAGA | Reverse primer for fragment 6 within *ilv1* locus |
| SD118 | GGAGCACGAACGCATTTAGTTAG | Forward primer for fragment 7 within *ilv1* locus |
| SD119 | GAATCTGTGGGTACTCGTTGTAAT | Reverse primer for fragment 7 within *ilv1* locus |

| | | |
|---|---|---|
| SD339 | GTCATTACAACGAGTACCCACAGA | Forward primer, checking for downstream of *ilv1* operon |
| SD340 | CCACTTCTTCAATTAACGTACATTCTTC G | Reverse primer, checking for downstream of *ilv1* operon |
| SD120 | GAAACCAGGAAGTGTGACGAAAC | Forward primer for fragment 1 within *ilv2* locus |
| SD121 | TGGCCTAGGCTCTAGTTTGTATC | Reverse primer for fragment 1 within *ilv2* locus |
| SD122 | GAAAGGATTAAGAGCGACAAATGCA | Forward primer for fragment 2 within *ilv2* locus |
| SD123 | GCGGAGATTCTCTTCTACTTCTGC | Reverse primer for fragment 2 within *ilv2* locus |
| SD124 | CGAGTACTTACAGAAATTCAGCAAGG | Forward primer for fragment 3 within *ilv2* locus |
| SD125 | ATACGAAGTGCAGCCATCATCATAC | Reverse primer for fragment 3 within *ilv2* locus |
| SD126 | CGACCAGAACCGAAAGTGAAAAC | Forward primer for fragment 4 within *ilv2* locus |
| SD127 | CAACCGGTTTCTCCATATCATGC | Reverse primer for fragment 4 within *ilv2* locus |
| | | |
